# Increased semaphorin, neuropilin, and plexin expression plays a role in recovery after traumatic brain injury

**DOI:** 10.1007/s11011-026-01788-x

**Published:** 2026-02-16

**Authors:** Aslı Okan, Zeynep Yılmaz Şükranlı, Taha Berkay Bor, Ali İmran Berkyürek, Serpil Taheri, Züleyha Doğanyiğit

**Affiliations:** 1 Department of Histology and Embryology, Faculty of Medicine, Yozgat Bozok University, Yozgat, 66100 Turkey; 2 Betul Ziya Eren Genome and Stem Cell Center, Kayseri, 38280 Turkey; 3 Department of Medical Biology, Faculty of Medicine, Erciyes University, Kayseri, 38039 Turkey

**Keywords:** Traumatic brain injury, Semaphorin, Neuropilin, Plexin, Mice

## Abstract

One of the leading causes of death and disability globally, affecting people of all ages, is traumatic brain injury (TBI). Damage to neural tissues associated with TBI can be classified into two categories: primary damage caused by direct mechanical forces resulting from trauma and secondary damage characterized by long-term neuroinflammation resulting from the molecular response of tissues and cells following primary injury. Although the effects of semaphorin (Sema) molecules on neural repair and regeneration are known, their role in the secondary injury process after TBI remains unclear. Sema proteins bind to their receptors, members of the neuropilin and plexin protein families, to carry out their actions. In this study, after mild TBI (mTBI) was induced, an acute mTBI group (observed for 24 h), a short-term mTBI chronic group, a long-term mTBI chronic group, and a sham group were created. As a result, neuronal damage is increased in acute and chronic mTBI models. Males exhibit increased levels of Sem-3 F, Plexin-A1, Neuropilin-1, and TNF-α protein expression in the hippocampus, particularly following chronic short-term mTBI. In addition, the chronic long-term group presented greater neuropilin 1 gene expression in the hypothalamus, hippocampus, pituitary, and hypophysis. Our findings suggest that semaphorin, neuropilin, and plexin proteins may help create new treatment techniques on the basis of their potential involvement in establishing and recovering from primary and secondary damage following TBI.

**Graphical Abstract:**

Males exhibit increased levels of Sem-3F, Plexin-A1, Neuropilin-1, and TNF-α protein expression in the hippocampus, particularly following chronic short-term mTBI. In addition, the chronic long-term group presented greater neuropilin 1 gene expression in the hypothalamus, hippocampus, pituitary, and hypophysis. Our findings suggest that semaphorin, neuropilin, and plexin proteins may contribute to the development of new treatment techniques based on their potential role in establishing and recovering from primary and secondary damage following traumatic brain injury.

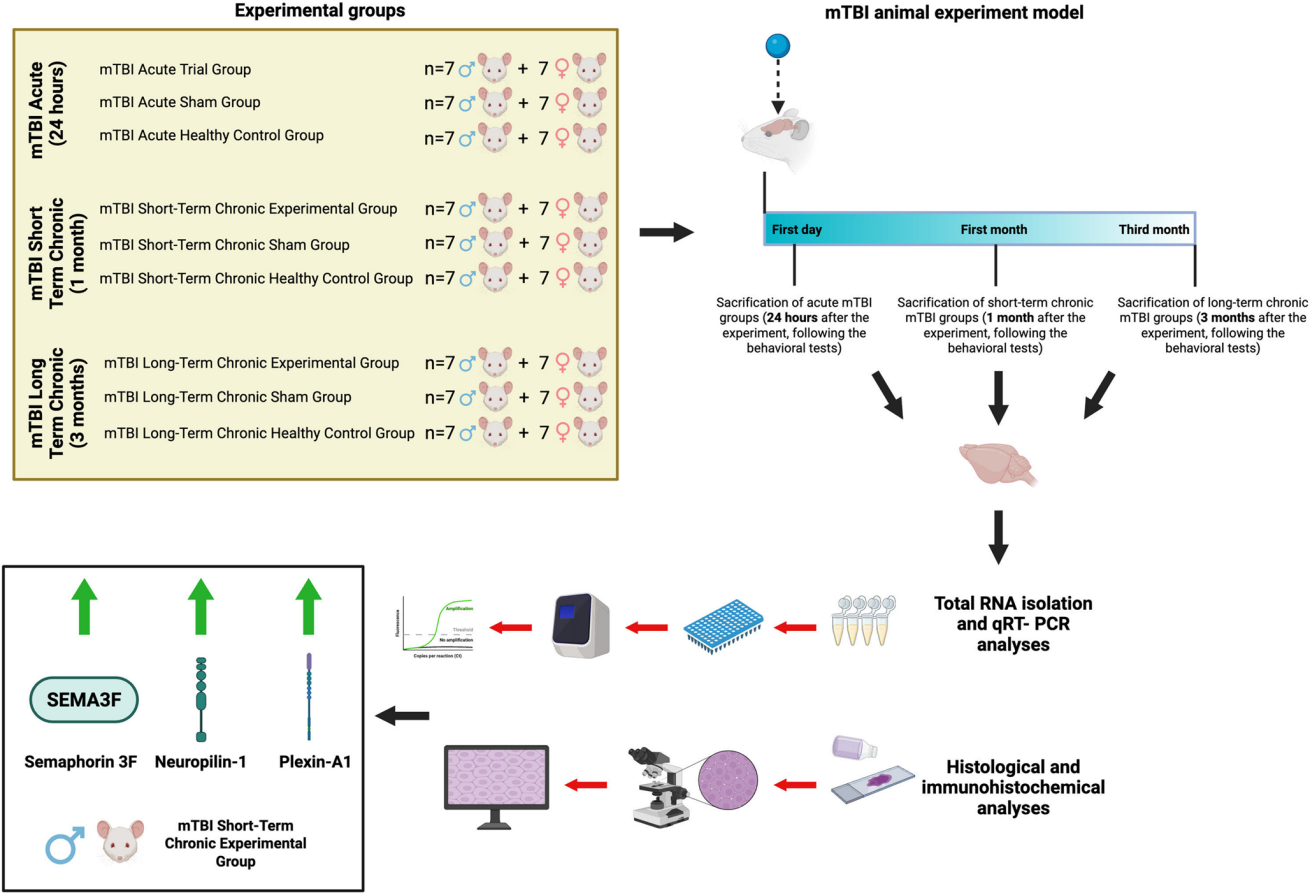

**Supplementary Information:**

The online version contains supplementary material available at 10.1007/s11011-026-01788-x.

## Introduction

One of the leading causes of death and disability globally, affecting people of all ages, is traumatic brain injury (TBI) (Dewan et al. 2019). TBI is classified into three severity groups according to severity: mild, moderate, and severe, and the most common type of TBI is mild TBI (mTBI). Damage to neural tissues associated with TBI can be divided into two categories: primary damage caused by direct mechanical forces as a result of trauma and secondary damage characterized by long-term neuroinflammation that occurs as a result of the molecular response originating from tissues and cells following primary injury (Ng and Lee 2019). Primary damage in TBI usually includes early injuries and necrotic cell death, and its treatment is aimed mainly at stabilizing the injury site and protecting against secondary damage. However, secondary damage is a result of molecular responses rather than being their cause. Mechanistically, cellular overstimulation causes secondary damage, which includes molecular responses such as mitochondrial dysfunction, oxidative stress, lipid oxidation, neuroinflammation, axon degeneration, and apoptotic cell death (Kaur and Sharma 2018). However, the precise mechanisms that trigger secondary damage remain incompletely understood.

Semaphorins (Sema) are cell membrane glycoproteins used in molecular studies to elucidate the recovery process after TBI because of their effects on neural repair and regeneration. Sema plays a key role in a variety of developmental processes that influence the nervous system, including synapse formation, cell migration, and neuronal proliferation. Sema expression, however, occurs following development (Carulli et al. 2021). Sema3A is a component of “perineuronal nets”, which are extracellular matrix structures surrounding neuronal cell types in the adult central nervous system (CNS), contributing to the closure of the critical period for plasticity (Boggio et al. 2019). Sema functions by binding to its receptors, the neuropilin (Npn) and plexin (Plxn) protein families (Takahashi et al. 1999). In the rodent hippocampus, the Npn1-PlxnA4-Sema3A receptor complex is known to mediate contextual memory formation via synaptic interactions (Jitsuki-Takahashi et al. 2021). Sema-Plxn signaling, particularly Sema2b and PlxnB signaling, has been reported to be important for the stabilization of synaptic transmission throughout the developing and adult nervous systems (Orr et al. 2017).

Sema molecules are also thought to be involved in the etiology of neurological degeneration-related diseases such as dementia, Alzheimer’s disease, multiple sclerosis, and stroke due to secondary damage that occurs in the chronic period after TBI. The expression of Sema3s in the central nervous system after trauma may be crucial for the early and long-term repair of injured tissue. In addition, there is an increase in Sema3A mRNA expression after lesions penetrate the CNS after trauma, which becomes more pronounced 1 day after axonal damage and 7 days after axonal damage and continues for up to 2 months (Pasterkamp et al. 1999). Another study conducted in 2019 revealed that increased Sema3A expression after TBI triggers secondary blood‒brain barrier damage. However, it has been reported that transfection with Sema3A-specific microRNAs (miRNAs) in mouse models can reduce this damage (Yang et al. 2019). Although the literature has shown different effects of Sema molecules on the recovery process in the brain after stroke, their roles in the recovery process after TBI have not yet been elucidated. The possible role of Sema in the molecular basis of the myelin sheath and neuroinflammation in secondary damage after TBI should not be ignored.

In our study, for the first time, we investigated the changes in the protein and gene expression of semaphorin, neuropilin, and plexin in brain tissue. To achieve this goal, we established the following experimental groups: acute mTBI (observed for 24 h), short-term chronic mTBI (observed for 1 month), and long-term chronic mTBI (observed for 3 months), along with corresponding sham groups (acute, short-term chronic, and long-term chronic). Using RT‒PCR, we examined the mRNA expression levels of semaphorin, neuropilin, and plexin in the hypothalamus, pituitary, hippocampus, and prefrontal cortex during both the acute and chronic phases of TBI. We aimed to investigate the relationship between the changes in the expression of the proteins encoded by these genes and the level of recovery by determining the changes in the expression of these genes in both the acute and long-term stages following TBI via immunohistochemistry and to evaluate clinical progression in future studies.

## Materials and methods

### Animals

Male and female BALB/c mice weighing 30–40 g and two months of age were randomly utilized in this investigation. The mice were obtained from the Betül-Ziya Eren Genome and Stem Cell Center’s Transgenics Department, Turkey. At two months of age, head trauma was induced according to the Marmarau trauma model (Tweedie et al. 2013). The experimental animal study was conducted with the approval of the local ethics committee of Erciyes University (HADYEK) (number 21/225).

### TBI model

Before trauma, local anesthesia was applied to the subcutaneous tissue of the mouse heads. To create mild trauma, the scalp of the anesthetized mouse was prepared by opening it with a scalpel, and the mouse was placed on the Marmarau trauma device. Under anesthesia, a plastic disk was placed on the surgically opened head of the mouse and placed under this tube. A 30-g weight was subsequently left from the top of the tube (Tweedie et al. 2013). This procedure was performed once to create mild head trauma. Then, for the mTBI acute group, the mice were sacrificed 24 h after the operation; for the mTBI short-term chronic group (Ge et al. 2018), the mice were sacrificed 1 month after the operation; and for the mTBI long-term chronic group (McInnes et al. 2019), the mice were sacrificed 3 months after the operation, and the relevant tissues were collected. For the sham groups, under the same conditions as those used for the trauma group, the scalp was opened, and no surgical procedure was performed. Only surgical sutures were placed. Then, for the mTBI acute sham group, the mice were sacrificed 24 h after the operation; for the mTBI short-term chronic group, the mice were sacrificed 1 month after the operation; and for the mTBI long-term chronic sham group, the mice were sacrificed 3 months after the operation, and the relevant tissues were collected. Since each group was terminated at different times and a common healthy control group could not be created, separate healthy control groups were created for the 3 main experimental groups.

### Animal grouping

The nine groups were divided into control, sham, and experimental groups, as indicated in Table 1.

**Table 1:**
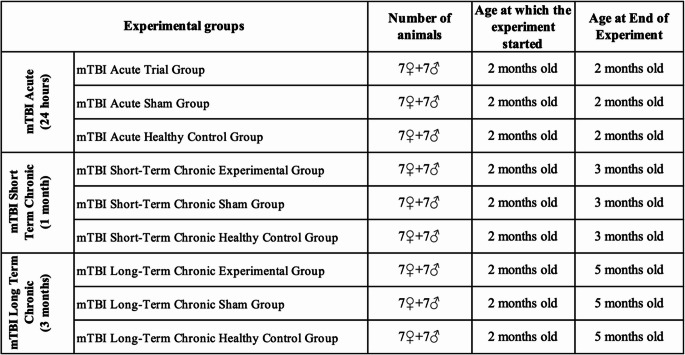
Experimental design of the study

### Behavioral experiments

After a model for mTBI chronic short-term (1 month) and mTBI chronic long-term (3 months) mice with traumatic brain injury and healthy control group mice were created, novel object recognition (Yilmaz Sukranli et al. 2024), tail suspension (Moy et al. 2008), marble test (Nicolas et al. 2006) social interaction test (Ikeda et al. 2013) and open field test (Silvero-Isidre et al. 2018) behavioral experiments were performed.

In this study, the early acute phase was defined as the period in which the initial pathophysiological and molecular alterations occur following trauma (Lu et al. 2025). To specifically investigate these early acute changes, mice in the acute TBI group were sacrificed 24 h after injury induction, and no behavioral tests were performed in this group. A total of five different behavioral experimental analyses were conducted in the study. Performing behavioral tests in the acute group would have shifted the assessment into the sub-acute phase (Erdman et al. 2011), which would not align with the primary objective of capturing early-phase alterations. Therefore, the acute group was evaluated exclusively at the molecular and histological levels to characterize the immediate effects of mild TBI.

### Total RNA isolation

After the behavioral experiments were completed, the mice were sacrificed, and tissues from the hypothalamus, pituitary, hippocampus, and prefrontal cortex were removed. The collected tissue samples were placed in 500 µL of QIAzol Lysis Reagent Isolation Reagent (Qiagen, Cat No: 79306 USA). Total RNA isolation was then performed according to the manufacturer’s instructions.

### cDNA Preparation and quantitative real-time polymerase chain reaction (qRT‒PCR)

Complementary strand DNA (cDNA) was obtained via the iScript™ cDNA Synthesis Kit (Bio-Rad, Cat No: 1708891, USA) according to the manufacturer’s protocol to study gene expression in RNA samples from the study group. The mRNA expression levels of *Sema3a*, *Nrp1*, and *Plxna1* were quantified from the obtained cDNA samples via the Roche Light Cycler LC480 system and the SsoAdvanced Universal SYBR Green Supermix (Bio-Rad, Cat No: 1725271, USA) kit. The primer sequences and the Roche Light Cycler LC 480 II Real-Time PCR Program used in the study are shown in Supplementary Table 1.

### Histologic analysis

The total brain samples of each group (five females and five males) were fixed with formaldehyde solution (10%), washed in tap water (1 night), and then dehydrated by passing the tissues through an increasing alcohol series. Then, the tissue samples were made transparent by leaving them in xylol (Merck, 108297), and after passing through the paraffin series, they were embedded in clean paraffin. A microtome was used to cut 5 $\:\mu\:$m thick slices of paraffin-embedded brain tissue samples, which were then stained with Harris hematoxylin (BES LAB, BS-001) and Eosin Y (BES LAB, BS-009). A Zeiss Axiscope 3 light microscope was used for histomorphological analysis, and a Colibri 3 digital camera was used for imaging (Doğanyiğit et al. 2023). The brain tissues of the experimental groups were examined for signs of neuronal degeneration and inflammatory cell infiltration in the cortex and hippocampus. Neuron degeneration was defined as eosinophilic neurons with pyknotic nuclei, cell enlargement, or shrinking (Tambe et al. 2016). Histopathological results in each category were scored as 0 = absent, 1 = mild, 2 = moderate, and 3 = severe. Quantification was performed randomly and blindly by two investigators. Ten mice (five females and five males) per experimental group were used for histomorphological scoring, with a minimum of 10 photos per mouse.

### Immunohistochemistry analysis

Anti-Semaphorin-3 F (ASR-056, Alomone labs, RRID: AB_2876826), anti-Plexin-A1 (APR-081, Alomone labs, RRID: AB_2756765), anti-Neuropilin-1 (ANR-063, Alomone labs, RRID: AB_2756695) and TNF-α (E-AB-22159, Elabscience) immunoreactivities were detected via immunohistochemical analysis in sections taken from the brain tissues of the experimental groups via the avidin-biotin peroxidase method (Okan et al. 2024). In summary, citrate buffer (pH: 6.0; Thermo Fischer Scientific, UK, AP-9003-500) was utilized to expose epitopes following the deparaffinization of 5 μm thick sections. The slides were subsequently immersed in a 3% hydrogen peroxide solution mixed with methanol to inhibit endogenous peroxidase activity. The Ultra V blocking solution (Thermo Fischer Scientific, UK, TA-125-UB) prevented nonspecific staining. Then, the samples were incubated with primary antibodies (dilution ratios of semaphorin-3 F (1:100), plexin-A1 (1:100), neuropilin-1 (1:50) and TNF-α (1:100) at 4 °C overnight. A biotinylated goat anti-polyvalent secondary antibody (Thermo Fischer Scientific, UK, TP-125-BN) was then added and incubated for 40 min at 37 °C. Following multiple washes with PBS, the mixture was incubated for 30 min at 37 °C with streptavidin peroxidase (Thermo Fischer Scientific, UK, TS-125-HR). The diaminobenzidine (DAB) chromogen (Thermo Fischer Scientific, UK, TA-125-HD) was used to visualize the antibody complex. After that, Gill III Hematoxylin (Merck, Germany, 1.05174.1000) was used to counterstain the sections. After they were subjected to a sequence of increasing alcohols to dehydrate them, they were sealed with Entellan (Merck, 1.07961). For each experimental group, six mice (three females and three males) were used for immunohistochemical analysis. A Zeiss Axiscope 5 Colibri 3 light microscope was used to examine the sections. ImageJ version 1.46 (National Institutes of Health, Bethesda, Maryland) was used to measure the levels of immunoreactivity.

### Analysis of statistics

To execute all the statistical tests and create graphs, GraphPad Prism software (version 8.4.3, 2020) was used. Statistical analyses were performed using two-way ANOVA, followed by Tukey’s multiple comparison test to compare the female experimental groups among themselves and the male experimental groups within their respective categories. In addition, Šidák’s multiple comparison test was applied to evaluate potential sex-related differences between the corresponding male and female experimental groups.

Group differences were deemed significant when they were *p* < 0.05.

## Results

### Behavioral experiment findings

After a model with mTBI chronic short-term (1 month) and mTBI chronic long-term (3 months) mice with traumatic brain injury and healthy control group mice were created, novel object recognition (Yilmaz Sukranli et al. 2024), tail suspension (Moy et al. 2008), marble tests (Nicolas et al. 2006), social interaction experiments (Ikeda et al. 2013) and open field tests (Silvero-Isidre et al. mamaktadır, 2018) were performed. During data analysis, each group was first compared with its respective sham and healthy control groups to determine whether there were significant differences between the trauma groups and their age-matched controls. Additionally, comparisons were made among the experimental groups (mTBI acute, mTBI chronic short-term (ST), and mTBI chronic long-term (LT)) to molecularly, phenotypically, and histologically assess which injury phase in the traumatized groups was associated with the most significant prognosis.

Behavioral data were only analyzed for the chronic groups, as no behavioral experiments were conducted on the mTBI acute group. This decision was made because animals participate in behavioral experiments only once per day. Given that the experiments would span one week and that the traumatic condition would persist into the subacute and chronic phases, reliable and distinguishable findings would not be obtained from the mTBI acute group. As a result, behavioral experiments and analyses were not performed on the mTBI acute group. The results of the behavioral experiments are displayed in Supplementary Table 2.

### Neuronal damage increases in acute and chronic mTBI models

In terms of neuronal degeneration, there was no discernible difference between the sexes in the findings obtained throughout the entire brain, particularly in the hippocampus.

**Fig. 1:**
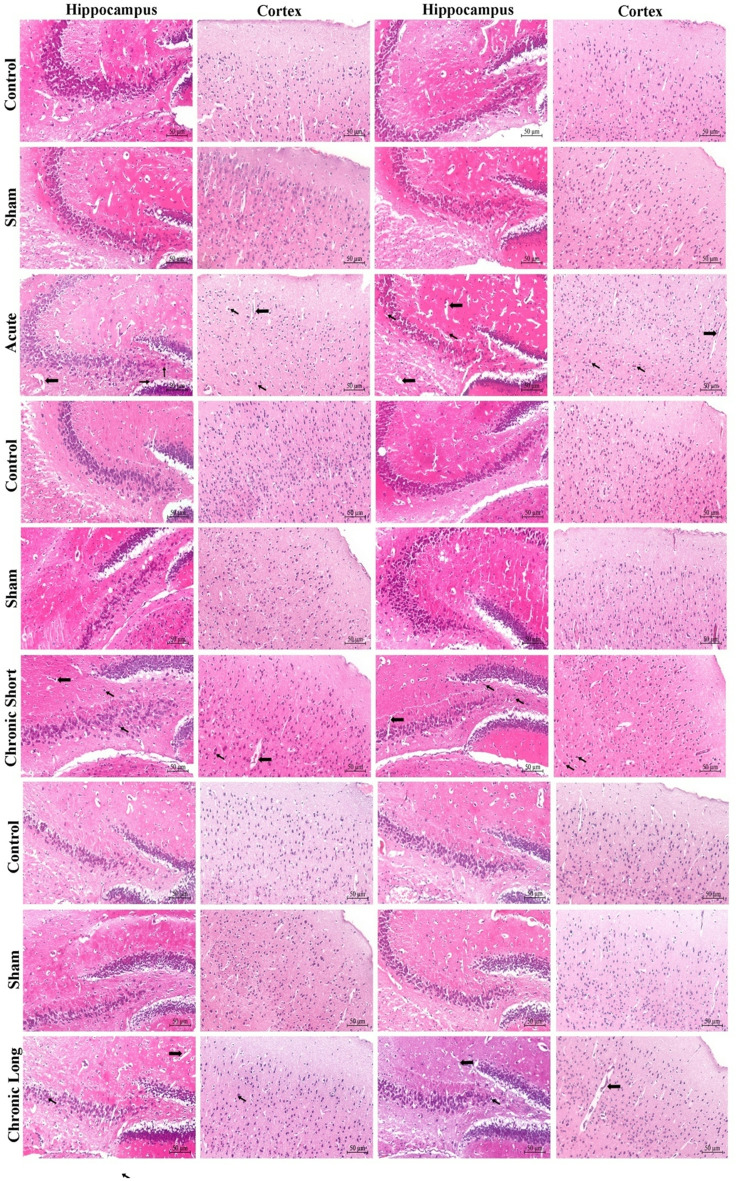
Hematoxylin and eosin (H&E)-stained images of total brain tissue from the hippocampus and cortex of the experimental groups. Images of the control (acute), sham (acute) and mTBI acute, control (chronic short), sham (chronic short) and mTBI chronic short groups and the control (chronic long), sham (chronic long) and mTBI chronic long groups. The thick black arrow shows vascular dilatation, and the thin black arrow shows neuron degeneration. Magnification 20X, scale bar 50 μm

When vascular dilatation was compared only between the mTBI acute female and male groups, it was found that males had more dilated blood vessels (Fig. 1; Table 2). Neuronal degeneration was more severe in the mTBI acute, mTBI chronic short-term, and mTBI chronic long-term groups than in the control and sham groups (Fig. 1). The average number of dilated blood vessels was greater in the mTBI acute male, mTBI chronic long-term male, and mTBI chronic long-term female groups than in the other groups (Table 2). In the evaluation of the prefrontal cortex areas of total brain tissue, a significant difference in terms of neuron degeneration was observed between the long-term mTBI female and male groups. The percentage of degenerated neurons was greater in males than in females. However, neuron degeneration significantly increased in the mTBI acute and chronic groups (Table 3). A comparison of the male and female experimental groups revealed that there was no difference in vascular dilatation between the sexes. Vascular dilatation did not differ across the female experimental groups when they were compared within themselves. Compared with the control and sham groups, the male mTBI acute and male mTBI chronic long-term groups presented more vascular dilatation (Fig. 1).

**Table 2:** Rates of hippocampal damage in the brain tissues of the experimental groups

Groups	Neuron Degeneration	Vascular Dilation
Control Female (Acute)	0,46$\:\pm\:$0,11	0,24$\:\pm\:$0,05
Sham Female (Acute)	0,4$\:\pm\:$0,18	0,44$\:\pm\:$0,16
mTBI Acute Female	1,04$\:\pm\:$0,26^ab^	0,38$\:\pm\:$0,08
Control Female (Short-Term)	0,3$\:\pm\:$0,10^c^	0,24$\:\pm\:$0,08
Sham Female (Short-Term)	0,44$\:\pm\:$0,05^c^	0,44$\:\pm\:$0,11
mTBI Chronic Female (Short-Term)	0,74$\:\pm\:$0,05^bd^	0,34$\:\pm\:$0,11
Control Female (Long-Term)	0,4$\:\pm\:$0,13^cf.^	0,46$\:\pm\:$0,05
Sham Female (Long-Term)	0,4$\:\pm\:$0,10^cf.^	0,5$\:\pm\:$0,15
mTBI Chronic Female (Long-Term)	1,02$\:\pm\:$0,13^abdegh^	0,78$\:\pm\:$0,16^acdf^
Control Male (Acute)	0,42$\:\pm\:$0,13	0,34$\:\pm\:$0,20
Sham Male (Acute)	0,3$\:\pm\:$0,15	0,28$\:\pm\:$0,08
mTBI Acute Male	1,02$\:\pm\:$0,14^jk^	0,82$\:\pm\:$0,25^jk^
Control Male (Short-Term)	0,56$\:\pm\:$0,24^l^	0,48$\:\pm\:$0,13
Sham Male (Short- Term)	0,4$\:\pm\:$0,10^l^	0,38$\:\pm\:$0,08^l^
mTBI Chronic Male (Short-Term)	0,94$\:\pm\:$0,18^jkmn^	0,4$\:\pm\:$0,1^l^
Control Male (Long-Term)	0,42$\:\pm\:$0,10^lo^	0,46$\:\pm\:$0,11^l^
Sham Male (Long-Term)	0,52$\:\pm\:$0,10^lo^	0,5$\:\pm\:$0,2
mTBI Chronic Male (Long-Term)	0,94$\:\pm\:$0,11^jkmnpr^	0,77$\:\pm\:$0,47^jkno^

Statistical comparisons were performed using two-way ANOVA with Sidak’s multiple comparison test. Data are expressed as mean ± SD. Superscripts indicate p < 0.05: female groups—a vs. control (acute), b vs. sham (acute), c vs. mTBI (acute), d vs. control (short-term), e vs. sham (short-term), f vs. mTBI (chronic short-term), g vs. control (long-term), h vs. sham (long-term); male groups—j vs. control (acute), k vs. sham (acute), l vs. mTBI (acute), m vs. control (short-term), n vs. sham (short-term), o vs. mTBI (chronic short-term), p vs. control (long-term), r vs. sham (long-term).

**Table 3:** Rates of damage to the brain tissues in the prefrontal cortex region in the experimental groups

Groups	Neuron Degeneration	Vascular Dilation
Control Female (Acute)	0,3$\:\pm\:$0,07	0,4$\:\pm\:$0,10
Sham Female (Acute)	0,46$\:\pm\:$0,15	0,52$\:\pm\:$0,10
mTBI Acute Female	1$\:\pm\:$0,33^ab^	0,38$\:\pm\:$0,08
Control Female (Short-Term)	0,36$\:\pm\:$0,08^c^	0,26$\:\pm\:$0,08
Sham Female (Short-Term)	0,44$\:\pm\:$0,13^c^	0,3$\:\pm\:$0,10
mTBI Chronic Female (Short-Term)	0,7$\:\pm\:$0,07^acd^	0,54$\:\pm\:$0,18
Control Female (Long-Term)	0,36$\:\pm\:$0,05^cf.^	0,38$\:\pm\:$0,13
Sham Female (Long-Term)	0,36$\:\pm\:$0,08^cf.^	0,52$\:\pm\:$0,08
mTBI Chronic Female (Long-Term)	0,66$\:\pm\:$0,15^acdgh^	0,5$\:\pm\:$0,10
Control Male (Acute)	0,36$\:\pm\:$0,15	0,24$\:\pm\:$0,11
Sham Male (Acute)	0,3$\:\pm\:$0,14	0,26$\:\pm\:$0,05
mTBI Acute Male	1,16$\:\pm\:$0,19^jk^	0,6$\:\pm\:$0,18^jk^
Control Male (Short-Term)	0,46$\:\pm\:$0,15^l^	0,3$\:\pm\:$0,07
Sham Male (Short- Term)	0,4$\:\pm\:$0,07^l^	0,42$\:\pm\:$0,10
mTBI Chronic Male (Short-Term)	0,86$\:\pm\:$0,20^jklmn^	0,46$\:\pm\:$0,05
Control Male (Long-Term)	0,38$\:\pm\:$0,04^lo^	0,42$\:\pm\:$0,10
Sham Male (Long-Term)	0,44$\:\pm\:$0,05 ^lo^	0,56$\:\pm\:$0,26
mTBI Chronic Male (Long-Term)	0,94$\:\pm\:$0,11^jkmnpr^	0,76$\:\pm\:$0,47^jkmnp^

Statistical comparisons were performed using two-way ANOVA with Sidak’s multiple comparison test. Data are expressed as mean ± SD. Superscripts indicate p < 0.05: female groups—a vs. control (acute), b vs. sham (acute), c vs. mTBI (acute), d vs. control (short-term), e vs. sham (short-term), f vs. mTBI (chronic short-term), g vs. control (long-term), h vs. sham (long-term); male groups—j vs. control (acute), k vs. sham (acute), l vs. mTBI (acute), m vs. control (short-term), n vs. sham (short-term), o vs. mTBI (chronic short-term), p vs. control (long-term), r vs. sham (long-term).

### In the hippocampus, sem-3 f, plexin-a1, and neuropilin-1 protein expression is increased in males, especially after chronic short-term mTBI

**Fig. 2:**
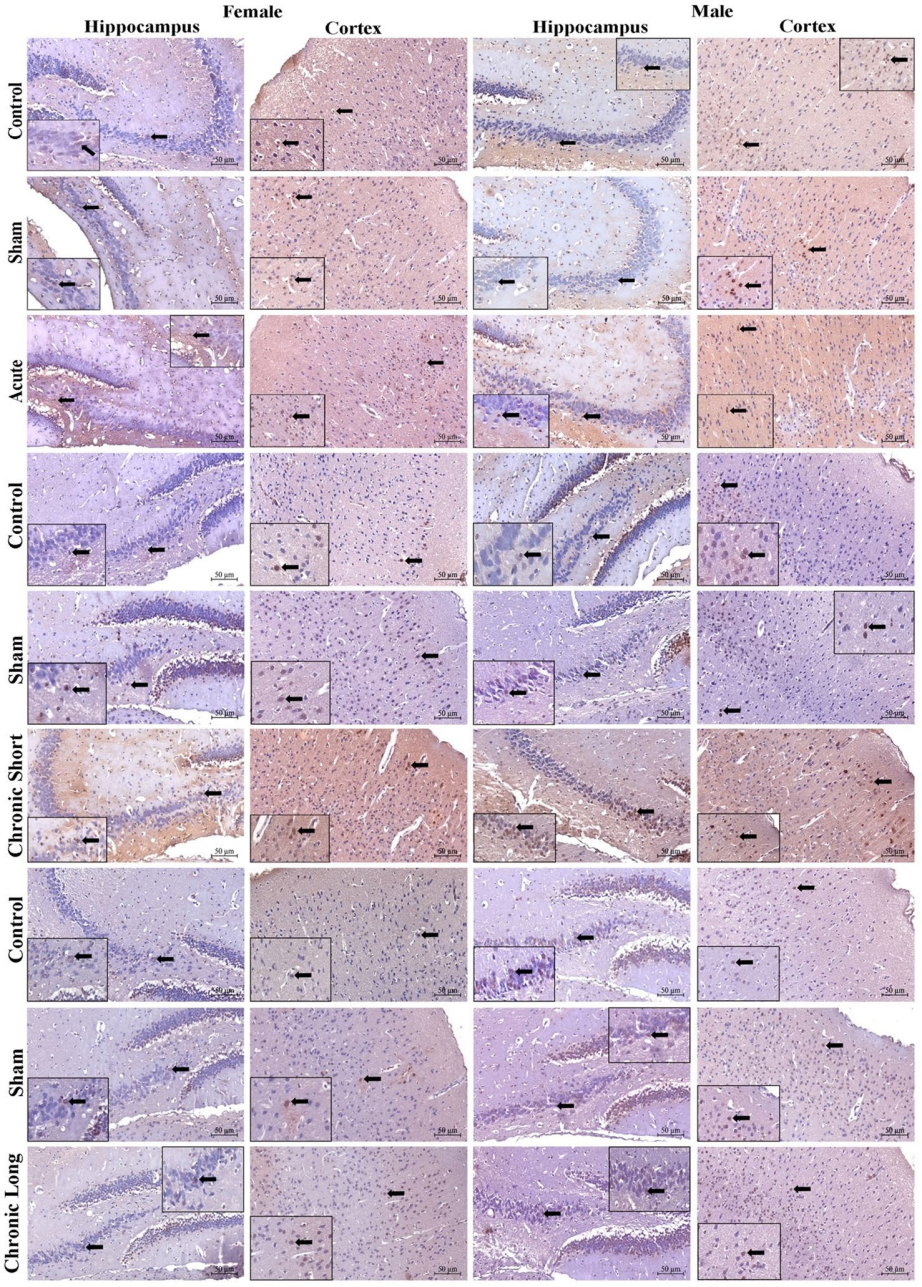
Semaphorin-3 F (Sema3F) immunoreactivity in the hippocampus and cortex across the experimental groups. Images of the control (acute), sham (acute), and mTBI acute groups; the control (short-term), sham (short-term), and MTBI chronic short-term groups; and the control (long-term), sham (long-term) and mTBI chronic long-term groups. Images were acquired from the hippocampus and cerebral cortex of the same brain level for each animal and are displayed at identical magnification and processing settings to permit visual comparison of staining intensity and cellular distribution. Black thick arrows indicate Sema3F-immunoreactive cells, allowing the reader to identify typical labeling patterns within each region and time window. All panels are shown at 20× objective; scale bar = 50 μm (applies to all images). The inset within each photomicrograph provides a higher-magnification view of the cellular region indicated by the arrow, illustrating the detailed morphology and localization of Sema3F-immunoreactive cells

**Fig. 3:**
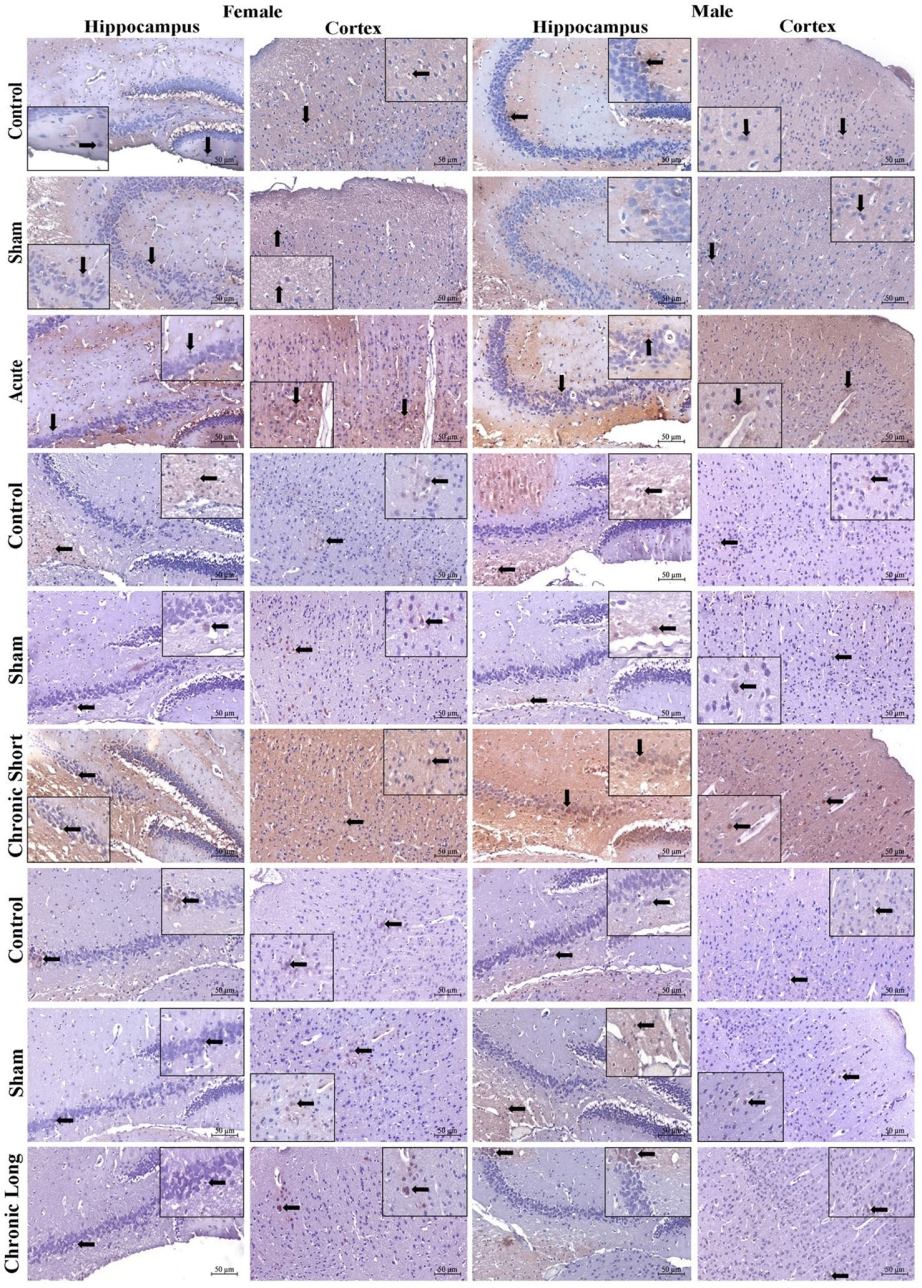
Plexin-A1 immunoreactivity in the hippocampus and cortex across the experimental groups. Images of the control (acute), sham (acute), and mTBI acute groups; the control (short-term), sham (short-term) and mTBI chronic short-term groups; and the control (long-term), sham (long-term) and mTBI chronic long-term groups. Images were acquired from the hippocampus and cerebral cortex of the same brain level for each animal and are displayed at identical magnification and processing settings to permit visual comparison of staining intensity and cellular distribution. The black thick arrow shows Plexin-A1 immunoreactive cells, allowing the reader to identify typical labeling patterns within each region and time window. All panels are shown at 20× objective; scale bar = 50 μm (applies to all images). The inset within each photomicrograph provides a higher-magnification view of the cellular region indicated by the arrow, illustrating the detailed morphology and localization of Plexin-A1 immunoreactive cells

**Fig. 4:**
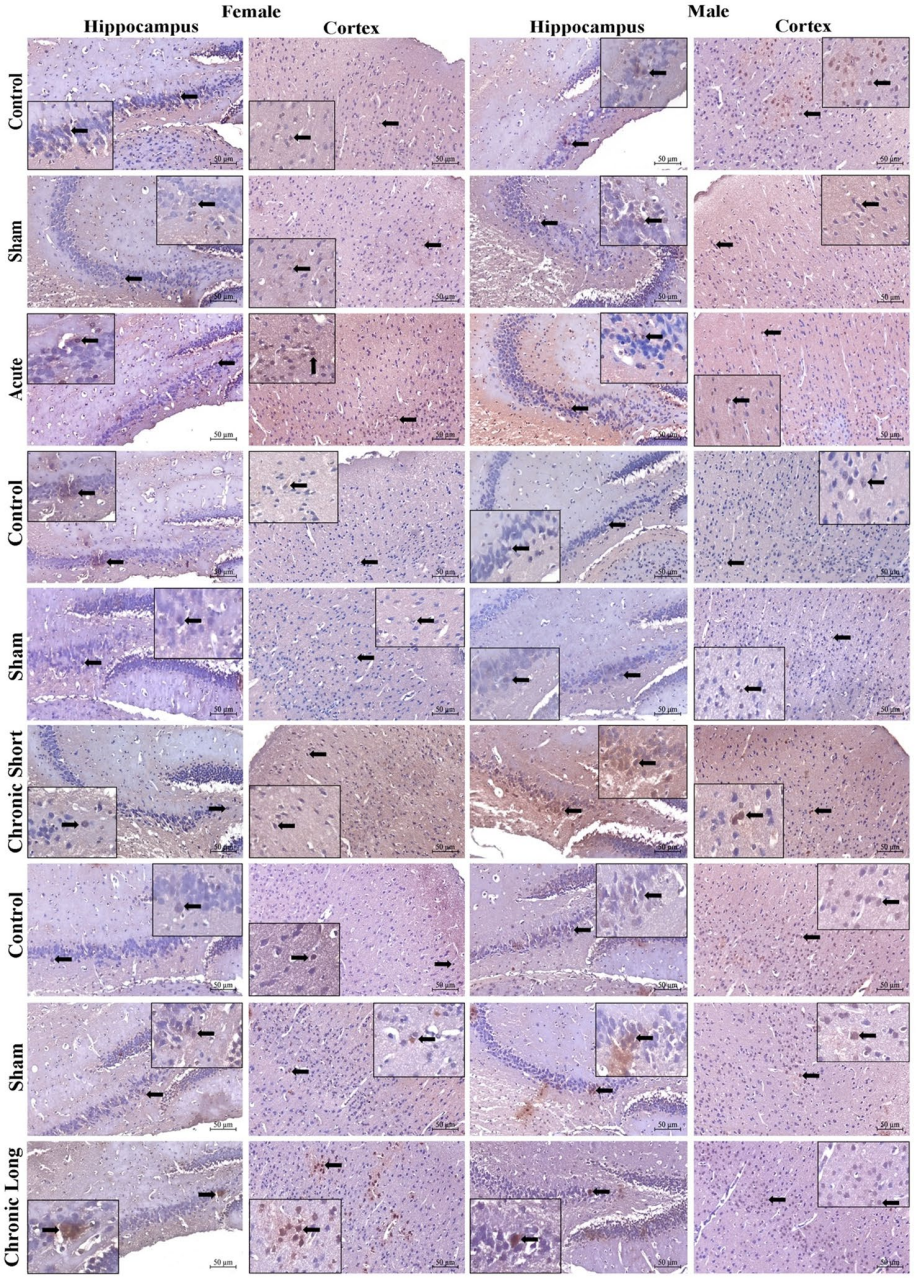
Neuropilin-1 immunoreactivity in the hippocampus and across the experimental groups. Images of the control (acute), sham (acute), and mTBI acute groups; the control (short-term), sham (short-term) and mTBI chronic short-term groups; and the control (long-term), sham (long-term) and mTBI chronic long-term groups. Images were acquired from the hippocampus and cerebral cortex of the same brain level for each animal and are displayed at identical magnification and processing settings to permit visual comparison of staining intensity and cellular distribution. The black thick arrow shows Neuropilin-1 immunoreactive cells, allowing the reader to identify typical labeling patterns within each region and time window. All panels are shown at 20× objective; scale bar = 50 μm (applies to all images). The inset within each photomicrograph provides a higher-magnification view of the cellular region indicated by the arrow, illustrating the detailed morphology and localization of Neuropilin-1 immunoreactive cells

**Fig. 5:**
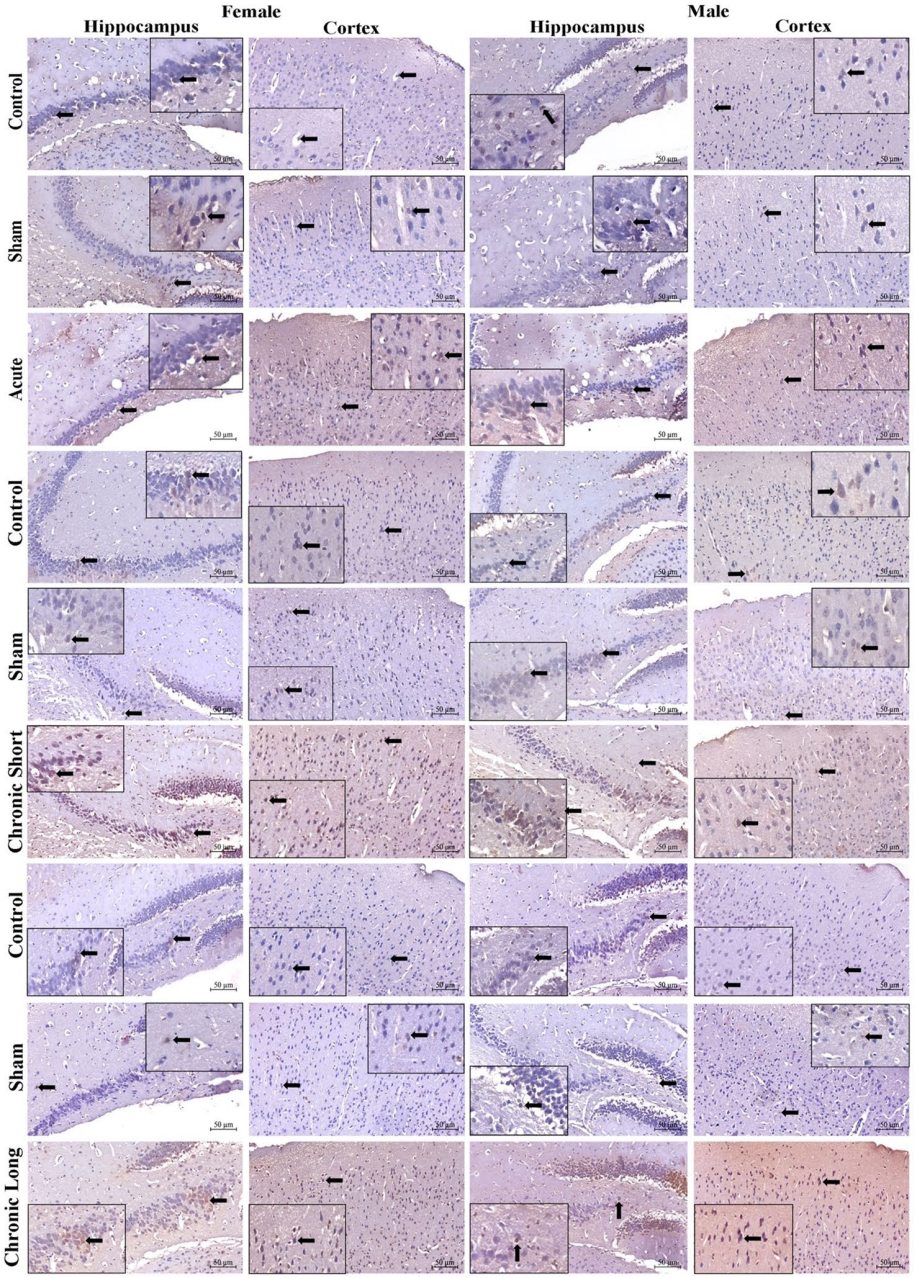
TNF-α immunoreactivity in the hippocampus and cortex across the experimental groups. Images of the control (acute), sham (acute), and mTBI acute groups; the control (short-term), sham (short-term), and mTBI chronic short groups; and the control (long-term), sham (long-term) and mTBI chronic long groups. Images were acquired from the hippocampus and cerebral cortex of the same brain level for each animal and are displayed at identical magnification and processing settings to permit visual comparison of staining intensity and cellular distribution. The black thick arrow shows TNF-α immunoreactive cells, allowing the reader to identify typical labeling patterns within each region and time window. All panels are shown at 20× objective; scale bar = 50 μm (applies to all images). The inset within each photomicrograph provides a higher-magnification view of the cellular region indicated by the arrow, illustrating the detailed morphology and localization of TNF-α immunoreactive cells

**Fig. 6:**
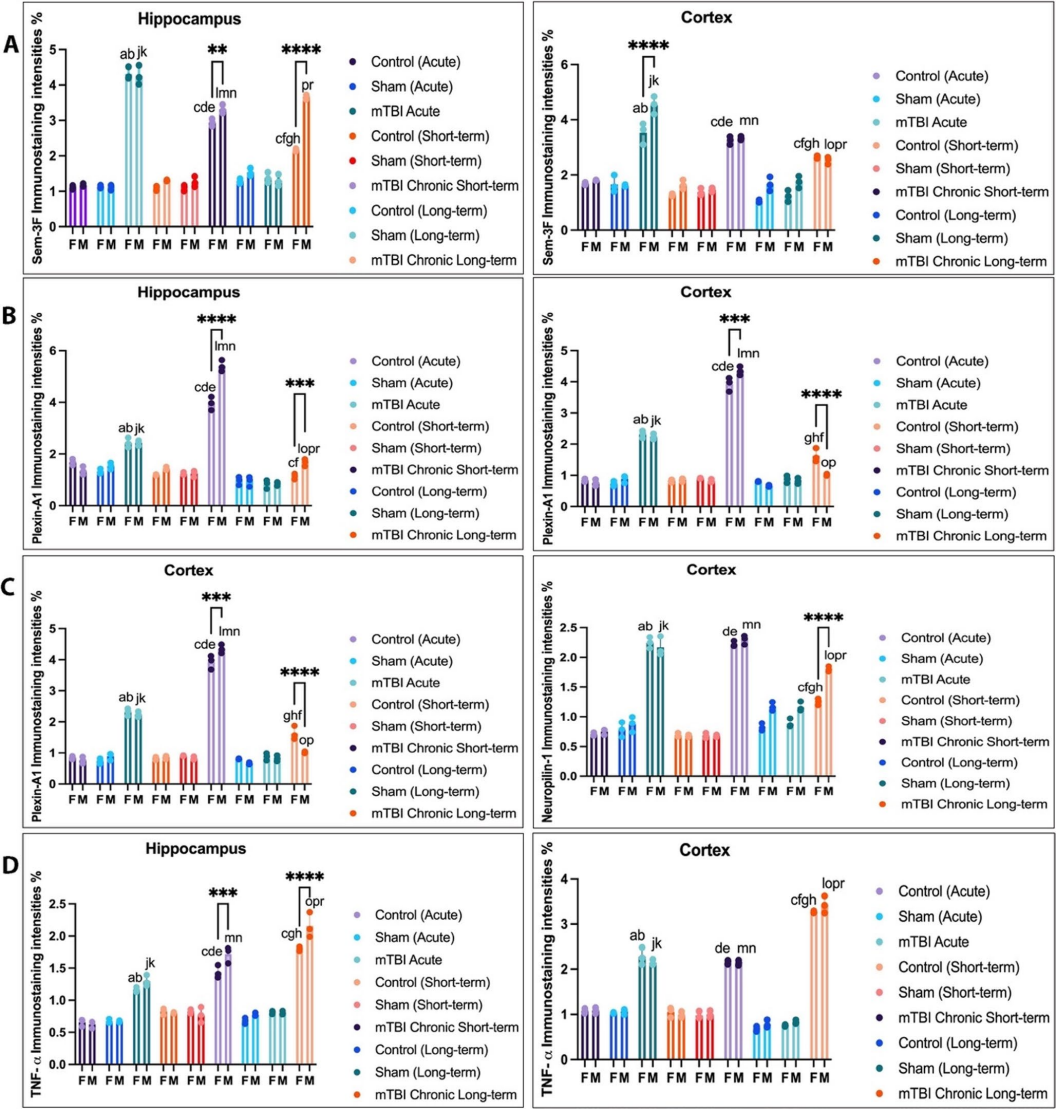
Separated scatter plots with bars illustrating the immunoreactivity density of Semaphorin-3 F (**A**), Plexin-A1 (**B**), Neuropilin-1 (**C**), and TNF-α (**D**) in the experimental groups. Each graph represents individual data points overlaid with mean ± SD values (separated scatter with bars). Statistical analyses were conducted using two-way analysis of variance (ANOVA) followed by Tukey’s and Šidák’s multiple comparison tests to evaluate within-sex and between-sex differences, respectively. In the female experimental groups, statistical significance was observed as follows: *a* *p* < 0.05 vs. control (acute); *b* *p* < 0.05 vs. sham (acute); *c* *p* < 0.05 vs. mTBI (acute); *d* *p* < 0.05 vs. control (short-term); *e* *p* < 0.05 vs. sham (short-term); *f* *p* < 0.05 vs. mTBI (short-term); *g* *p* < 0.05 vs. control (long-term); *h* *p* < 0.05 vs. sham (long-term). In the male experimental groups, significant differences were as follows: *j* *p* < 0.05 vs. control (acute); *k* *p* < 0.05 vs. sham (acute); *l* *p* < 0.05 vs. mTBI (acute); *m* *p* < 0.05 vs. control (short-term); *n* *p* < 0.05 vs. sham (short-term); *o* *p* < 0.05 vs. mTBI (short-term); *p* *p* < 0.05 vs.control (long-term); *r* *p* < 0.05 vs. sham (long-term)

Semaphorin 3 F (Sema-3 F), Plexin-A1, Neuropilin-1, and TNF-α expression increased in total brain tissues in all mTBI models. Sema-3 F was significantly increased in the cortex of the acute mTBI group (Figs. 2, , , , and 6). When we compared the sexes, we found that the expression of Sema-3 F increased in the male hippocampus in the mTBI chronic short-term and mTBI chronic long-term groups and increased in the male cortex in the mTBI acute group (Figs. 2 and 6, *p* < 0.05). Plexin-A1 (Figs. 3 and 6) and neuropilin-1 (Figs. 4 and 6) were significantly increased in the cortex of the mTBI chronic short-term groups. When we compared the sexes, the expression of Plexin-A1 in both the hippocampus and cortex increased in males in the mTBI chronic short-term and mTBI chronic long-term groups (*p* < 0.05). The expression of neuropilin 1 was increased in the hippocampus in the mTBI chronic short-term group of males, whereas it was increased in the cortex in the mTBI chronic long-term group of males (Figs. 4 and 6, *p* < 0.05). TNF-α expression increased more in the hippocampus, especially in males in the mTBI chronic short-term and long-term groups (Figs. 5 and 6).

### Neuropilin 1 gene expression in the hypothalamus, pituitary, hippocampus, and prefrontal cortex was greater in the chronic long-term group than in the control group

**Fig. 7:**
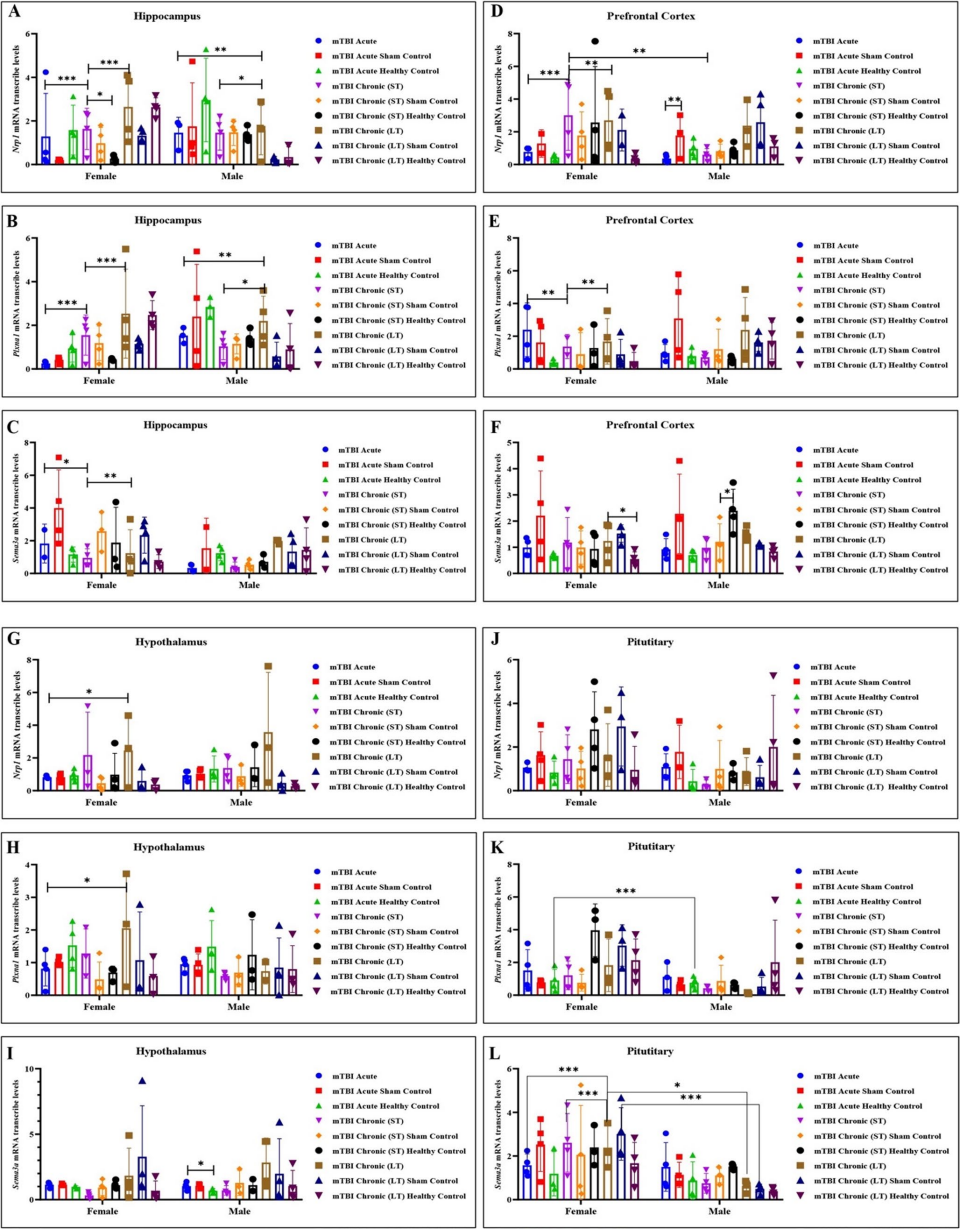
Sex-dependent alterations in ***Nrp1***,*** Plxna1***, and ***Sema3a*** gene expression in the hippocampus, prefrontal cortex, hypothalamus, and pituitary tissues between groups following mild traumatic brain injury (mTBI) (**A**–**C**) mRNA expression levels of *Nrp1*, *Plxna1*, and *Sema3a* genes in the hippocampus; (**D**–**F**) mRNA expression levels of *Nrp1*, *Plxna1*, and *Sema3a* genes in the prefrontal cortex; (G–I) mRNA expression levels of *Nrp1*, *Plxna1*, and *Sema3a* genes in the hypothalamus; and (**J**–**L**) mRNA expression levels of *Nrp1*, *Plxna1*, and *Sema3a* genes in the pituitary gland of female and male mice following acute, short term (ST) chronic or long term(LT) chronic phases of mTBI and their corresponding sham control and healthy control groups (Data are presented as mean ± SD (*N* = 4 per sex and group). Statistical analysis was performed using two-way ANOVA followed by appropriate post hoc multiple comparisons; *p* < 0.05*, *p* < 0.01**, *p* < 0.001***, and *p* < 0.0001, respectively, were considered statistically significant)

When the hippocampus of the trauma groups was compared, *Neuropilin 1* and *Plexin-A1* gene expression was high in the long-term mTBI group, whereas *Sema-3a* expression was low (Fig. 7A, B, C). In the prefrontal cortex, *neuropilin 1* and *Plexin-A1* levels were increased in the mTBI chronic short-term groups, whereas there was no difference in *Sema-3 A* gene expression between the groups. *Neuropilin 1* gene expression was greater in females than in males in the chronic short-term group (Fig. 7D, E, F). When the trauma groups were compared, the expression of the *Neuropilin 1*, *Plexin-A1*, and *Sema-3 A* genes in the hypothalamus was greater in the mTBI chronic long-term group than in the mTBI acute and short-term groups (Fig. 7G, H, I). While no difference in *Sema-3 A* expression according to sex was detected, the *expression of the neuropilin 1* and *Plexin-A1* genes was greatest in females in the long-term mTBI groups (Fig. 7G, H, I). While there was no difference in *Plexin-A1* or *Sema-3 A* expression in the pituitary between the trauma groups, the *neuropilin 1* gene was expressed at higher levels in the chronic short-term and long-term groups than in the mTBI acute group. *Sema-3 A* gene expression was greater in females than in males in the chronic long-term group and females in the other trauma groups (Fig. 7J, K, L).

## Discussion

TBI is known to be the health problem with the highest incidence among all neurological disorders. According to population-based epidemiological studies conducted in the United States (US) and Canada, the incidence of TBI is approximately 800–1000 per 100,000 people (Dewan et al. 2019; Langer et al. 2020; Taylor et al. 2017). TBI is not only an acute condition but also a chronic disorder with a risk of neurodegeneration in the future. Remyelination in the CNS requires the activation of oligodendrocyte precursor cells and their differentiation into mature oligodendrocytes. Many external factors, including the immune response, regulate the function of oligodendrocyte precursor cells in the damaged CNS. Sema3s are known to affect oligodendrocyte maintenance and remyelination (Kotter et al. 2011). Sema proteins, such as Sema3A and Sema3F, are increased in the demyelinated lesions of multiple sclerosis (MS) patients and in experimental animal models (Williams et al. 2007). Accordingly, Sema is associated with myelin damage, remyelination, and neuroinflammation processes. In addition, oligodendrocytes and their precursor cells express Npns (Npn1 and Npn2) and various Plxns. This expression profile indirectly indicates that these cell types are sensitive to Sema (Boyd et al. 2013; Syed et al. 2011). In line with the literature, according to the immunohistochemical results, the expression of Sem-3 F, neuropilin 1 (Npn1), and Plexin-A1 (PlxA1) was increased in the mTBI acute and mTBI chronic short groups compared with the mTBI chronic long group. Additionally, TNF-α, which plays important roles in neuroinflammation as a trigger and perpetuator of the neurodegenerative process (Clay Goodman et al. 1990), was elevated in the brains of all trauma groups. In our study, the increase in the expression of Sema3F and related proteins in both the early and chronic short phases compared with the chronic long phase after CNS trauma suggested that they may contribute to the healing process in the early stages.

The semaphorin family regulates synaptic transmission in the adult hippocampus (Sahay et al. 2005). Therefore, semaphorins secreted from the hippocampus have the potential to modulate neural circuit function. A 2024 study reported that TBI promotes neurogenesis in the adult hippocampus of male mice. Single-cell RNA sequencing and spatial transcriptomic methods revealed an increase in the number of neuronal cells derived from neural stem cells and a simultaneous decrease in the number of astrocyte cells derived from neural stem cells (Bielefeld et al. 2024). These findings suggest that the Sema family, especially Sema3A, may play a role in the cellular recovery process in brain tissue after injury. In addition, Sema3 and its related receptors have been shown to play a role in the development and maturation of hippocampal connections (Bagri and Tessier-Lavigne 2002; Mata et al. 2018). Sema3A may be one of the factors regulating axonal specialization in the hippocampus.

Furthermore, the hippocampus undergoes atrophy for weeks to months after TBI and shows deficits in long-term potentiation (LTP), a persistent increase in synaptic strength that is considered a model of learning and memory (Tomaiuolo 2004). The sensitivity of the hippocampus to TBI necessitates the determination of the mechanism of memory changes. The presence of Sema and related pathways may be of interest in determining hippocampal LTP and cell signaling deficits following TBI. Our findings revealed that, consistent with our immunohistochemistry findings, Sem3A gene expression increased in the chronic short-term group but decreased in the chronic long-term group. However, PlxnA1 and Npn1 gene expression in the hippocampus increased in both chronic groups but increased more in the chronic long-term group. In the cortex, Npn1 and PlxnA1 expression was increased in the chronic short-term group, whereas there was no difference in Sem3A gene expression between the groups. Npn1 gene expression was greater in females than in males in the chronic short-term group. These data suggest that Sem3A may contribute to the recovery process of dysfunction caused by chronic short-term hippocampal atrophy after TBI. This finding may also suggest that the hippocampus, after TBI, increases Npn1 and PlxnA1 expression to promote long-term cellular repair and plasticity.

Semas perform their functions by binding to their receptors, the Npn and Plxn protein families. In the rodent hippocampus, the neuropilin1-plexinA4-semaphorin3A receptor complex is known to mediate contextual memory formation through synaptic interactions (Jitsuki-Takahashi et al. 2021). There are not enough studies in the literature on the role of neuropilins in TBI. We recommend that Sema and related Npn/Plxn molecules, which are responsible for regulating axonal transmission in the brain, be further investigated in secondary injury mechanisms after TBI. Our findings revealed that Npn1 gene expression in the hypothalamus was greater in the chronic long-term group than in the sham and control groups, whereas Npn1 gene expression in the hypothalamus was lower in the acute TBI group than in the chronic long-term group. The increased Npn1 expression in the hypothalamus in the chronic long-term group suggests that TBI triggers the expression of this gene by increasing neuroinflammation in the long-term. Similarly, we reported that the gene expression of Npn1 in the pituitary gland decreased in the acute phase compared with that in the long- and short-term groups. The decrease in Npn1 in acute TBI may indicate that the pituitary gland develops a response to minimize damage together with the hypothalamus in the acute phase after TBI. In this context, long-term effects of TBI may be related to neurodegenerative processes, and Npn1 may play a potential regulatory role in these processes. In contrast, the decrease in Npn1 expression observed after acute TBI may indicate that Npn1 in the hypothalamus is regulated by a different mechanism in the acute phase.

In another study conducted with mice, the role of Sema3A in secondary blood‒brain barrier damage that develops due to traumatic brain injury was investigated. According to the findings obtained in this study, Sema3A plays a role in the development of secondary blood–brain barrier damage through the regulation of Npn1, PlxnA1, and miR-30b-5p, and the suppression of Sema3A after traumatic brain injury may be a potential treatment method (Yang et al. 2019). The findings of these studies and our research parallel each other: the Sema3A, Npn1, and PlxnA1 gene expression levels in the mTBI (chronic short- and long-term) groups were greater than those in the acute and control groups, and the expression levels increased with trauma.

The Sema, Npn1, and PlxnA1 expressions, which are associated with many neurological diseases, such as epilepsy, Alzheimer’s disease, autism, and multiple sclerosis, were investigated histologically and molecularly together for the first time in TBI and contributed to the literature in terms of elucidating the molecular mechanisms that play a role in the acute and chronic phases after TBI. The differences observed in our results between mRNA and protein levels are frequently seen due to the complex regulation of gene expression that extends beyond transcription. Post-transcriptional mechanisms, including differences in mRNA stability, alternative splicing, and regulation by microRNAs or RNA-binding proteins, can significantly influence translational efficiency and decouple protein abundance from transcript levels. Additionally, translational control and temporal delays between transcription and translation can result in transient mismatches between mRNA and protein expression. Protein abundance is further shaped by post-translational modifications and variable protein degradation rates mediated by proteasomal or lysosomal pathways. Consequently, the correlation between mRNA and protein levels is often moderate rather than linear, particularly under dynamic or stress-related biological conditions (Maier et al. 2009; Liu et al. 2016). In this study, to elucidate the molecular mechanisms underlying TBI and secondary damage that occurs in the brain after mTBI, data on the Sema, Npn1, and PlxnA1 genes and proteins were obtained from brain tissue. Although the findings are promising, more comprehensive studies are needed in the future to understand the molecular mechanisms more clearly.

## Limitation

A key limitation of this study is the necessity of using separate healthy control groups for each time point. Semaphorins, neuroplastin, and plexins play critical roles in neurodevelopmental processes, axon guidance, and synaptic plasticity, and previous studies have shown that the gene and protein expression levels of these molecules can change with aging. Because the acute mTBI group was sacrificed at 2 months of age, the short-term mTBI chronic group at 3 months, and the long-term mTBI chronic group at 5 months, using a single common healthy control group would have introduced age-related variability that could confound the interpretation of temporal changes following injury. Therefore, age-matched healthy control groups were used for each time point to ensure biologically accurate comparisons. Although this approach prevents direct comparison to a single baseline control set, it provides more reliable and meaningful interpretations by minimizing age-dependent bias.

## Supplementary Information

Below is the link to the electronic supplementary material.

ESM 1 (DOCX 19.2 KB)

ESM 2 (DOCX 24.1 MB)

## Data Availability

The data will be made available from the corresponding authors upon reasonable request.
