# Supplementary material for: Increased semaphorin, neuropilin, and plexin expression plays a role in recovery after traumatic brain injury
Source: Metab Brain Dis. 2026 Feb 16;41(1):35. doi: 10.1007/s11011-026-01788-x (PMC12909346; doi:10.1007/s11011-026-01788-x)
Supplement: Supplementary file 1 — (DOCX 19.2 KB) [file 11011_2026_1788_MOESM1_ESM.docx]

**Supplementary Table 1**

**Total RNA Isolation from Tissues**

500 µl Qiazol Lysis Reagent Isolation Reagent (Qiagen, Cat No: 79306 USA) was added to the tissue taken into 2 ml Eppendorf tubes and homogenized with a syringe. Then, 180 µl chloroform was added, vortexed for 15 seconds and incubated at room temperature for 5 minutes. Then, it was centrifuged at 12000 g for 20 minutes at +4°C and the aqueous phase was transferred to a new Eppendorf. Isopropanol as much as the aqueous phase was added and turned upside down several times. After incubating at room temperature for 10 minutes, it was centrifuged at 12000 g for 10 minutes at +4°C, the supernatant was discarded, 1 ml of 75% ethanol was added and vortexed. After centrifuging at 7500 g for 5 min at +4°C, the supernatant was discarded and the ethanol around the pellet was removed with a pipette and cleaned. Afterwards, it was dried at room temperature for 5-10 min and the pellet was resuspended with 50 µl of nuclease-free water. After resuspension, it was kept in a +4°C refrigerator for 10-15 min. After the obtained RNA sample was measured in the Nanodrop device and until all samples were studied, it was removed to -80°C.

**cDNA Synthesis**

To study gene expression from RNA samples obtained from the study group, the iScript™ cDNA Synthesis Kit (Biorad, Cat No:1708891, USA) was used to obtain complementary strand DNA (cDNA) from RNAs obtained from the study group, under the manufacturer's protocol.

**Table 1. Preparation of the mixture required for cDNA synthesis**

| Ingredients For each sample | Ingredients For each sample |
| --- | --- |
| Reaction Buffer(5X) 4 µl | Reaction Buffer(5X) 4 µl |
| Reverse Transcriptase 1 µl | Reverse Transcriptase 1 µl |
| dH_2_O 10 µl | dH_2_O 10 µl |
| RNA 4 µl | RNA 4 µl |

A reaction mixture was prepared for cDNA isolation with the amounts of reaction buffer, reverse transcriptase, and dH2O specified for each sample in **Table 1**. Then, 16 µl of this mixture was distributed for each sample. The relevant RNA samples were added to their relevant tubes. Then, the desired product was obtained by placing it in the Thermal Cycler device under the appropriate reaction conditions shown in **Table 2** for PCR.

**Table 2. PCR heater program**

| 25°C | 5 min. |
| --- | --- |
| 46°C | 20 min. |
| 95°C | 1 min. |
| 4°C | ∞ |

At the end of PCR, we have 20µl cDNA product. The cDNA samples obtained were diluted 1/5 with Nuclease Free Water (Qiagen, Germany).

**Determination of Gene Expression Levels in Real Time PCR**

The mRNA expression levels of Sema3a, Nrp1, and Plxna1 were determined separately from the obtained cDNA samples using the Roche Light Cycler LC480 device and the SsoAdvanced Universal SYBR Green Supermix (Biorad, Cat No: 1725271, USA) kit according to the following protocol. The reaction mix was prepared in the amounts specified in **Table 3** and distributed to the plate wells for each sample. The primers used in the experiment are given in **Table 4**.

**Table 3. Primers and sequences used in the experiment**

| **Gene Name** | **5'->3' Sequence** | **Amplicon Size (bp)** | **Tm** |
| --- | --- | --- | --- |
| ***Sema3a*** | **F:** AGCCCTTATGATCCCAAACTACTGA | 94 bp | 60 |
|  | **R:** CGAAGTCCCGTCCCATGAA |  |  |
| ***Nrp1*** | **F:** CTCTCCTTCCCGCAGACAAC | 252 bp | 59 |
|  | **R:** AGCTCCGATTTCCTCTCCCT |  |  |
| ***Plxna1*** | **F:** AAGCGGCCTACTCCCTTTTC | 292 bp | 59 |
|  | **R:** AGAATTCATGGCCCAGGGT |  |  |
| ***Actb*** | **F:** CACCATTGGCAATGAGCGGTTC | 135 bp | 60 |
|  | **R:** AGGTCTTTGCGGATGTCCACGT |  |  |
|  |  |  |  |

**Table 4. Preparation of reaction for gene expression**

| Sybr Green Master Mix | 10 ul |
| --- | --- |
| Primer F | 0,5 ul |
| Primer R | 0,5 ul |
| dH_2_O | 5 ul |

After distributing 16 ul of mixed plate into the wells, 4 ul of cDNA samples diluted at a ratio of 1:5 were added onto the musk. Then, the plate was covered with a transparent sealer. Air bubbles were eliminated by spinning and the PCR program given in **Table 5** was run on the Roche Light Cycler LC 480 II device and Ct values ​​were determined. β-Actin was used as the House-Keeping gene. The CT values ​​obtained at the end of the PCR were normalized to Ct values ​​using the 2-ΔΔCt method.

**Table 5. Roche Lightcycler LC480 II Real-Time PCR Program**

| Pre-incubation | 95 ^o^C | 10 min |  |
| --- | --- | --- | --- |
|  | 95 ^o^C | 10 sec | 45 cycle |
| Amplification | 60 ^o^C | 30 sec |  |
|  | 72 ^o^C | 1 sec |  |
| Cooling | 40 ^o^C | 30 sec |  |
