# Supplementary material for: Increased semaphorin, neuropilin, and plexin expression plays a role in recovery after traumatic brain injury
Source: Metab Brain Dis. 2026 Feb 16;41(1):35. doi: 10.1007/s11011-026-01788-x (PMC12909346; doi:10.1007/s11011-026-01788-x)
Supplement: Supplementary file 2 — (DOCX 24.1 MB) [file 11011_2026_1788_MOESM2_ESM.docx]

**1. Behavioral Experiment Findings**

**1.1. Novel Object Recognition Test Findings**

The novel object recognition test determined that the mTBI Chronic (CD) group, mTBI Chronic (CD) sham control group, and healthy control group showed more interest in the new object after trauma, but this difference was not statistically significant. It was found that only the mTBI chronic (CD) group showed statistically more interest in the new object than the healthy control **(Figure 1A).** When the changes were evaluated between the genders, no statistically significant difference was detected **(Figure 1B).**

When the interest in the new object of the mTBI chronic (CD), mTBI chronic (CD) sham control, and healthy control groups was measured; no significant difference was detected within the groups regarding interest in the new object and the old object. At the same time, no significant difference was observed between the genders **(Figure 1C-D).**

When it was investigated whether there was a significant difference between the experimental groups; No statistically significant difference was found between mTBI chronic (KD) and mTBI chronic (UD) in terms of interest in new objects and old objects. However, no statistically significant difference was found between genders. It is noteworthy that only mTBI chronic (UD) males showed more interest in old objects **(Figure 1 E-F).**

**
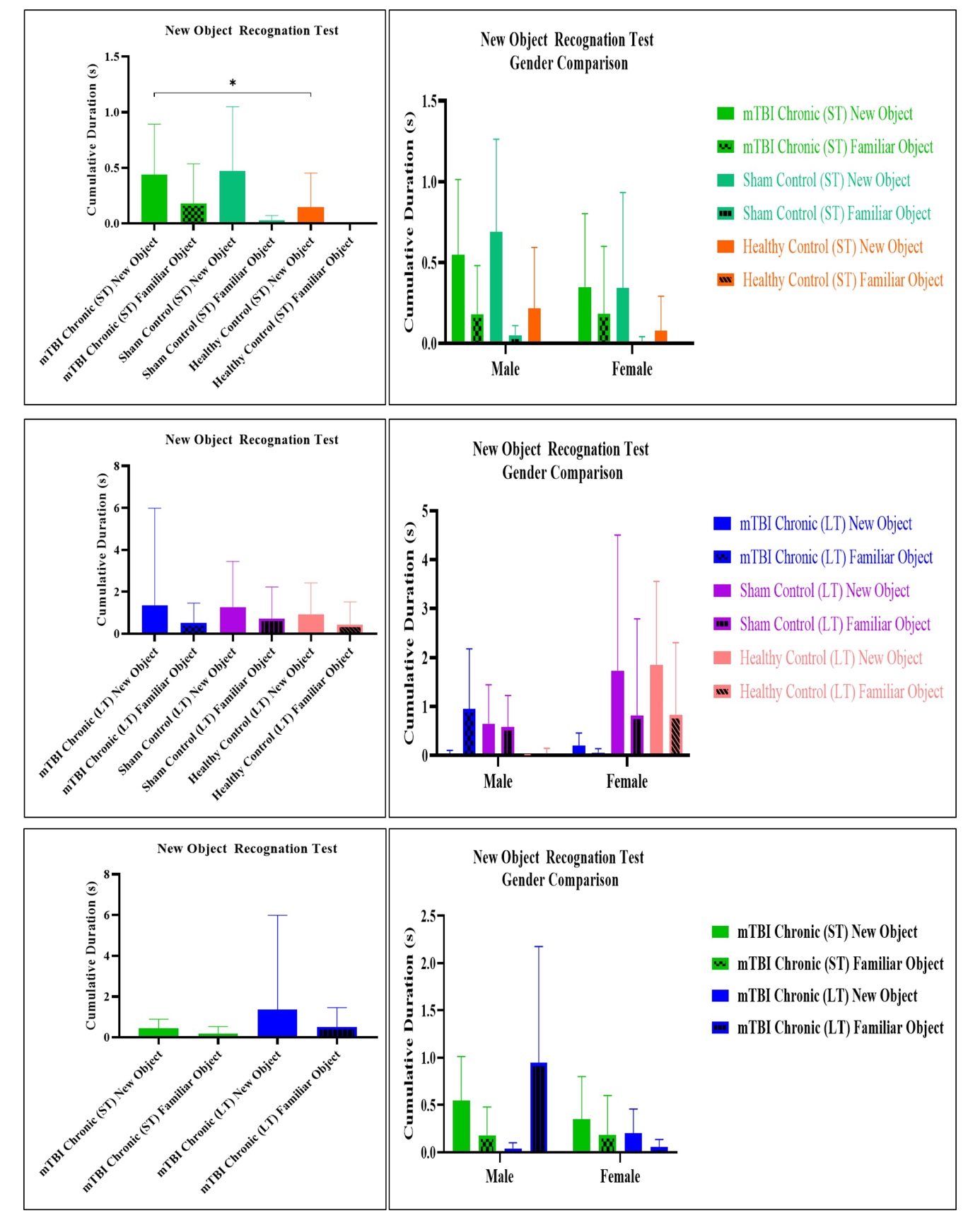
**

**Figure 1. Comparison of interest in new and old objects in a novel object recognition test.**

(A. Comparison of interest in new objects and old objects between the mTBI chronic short-term group and controls, B. Comparison of interest in new objects and old objects between the mTBI chronic short-term group and controls across genders, C. Comparison of interest in new objects and old objects between the mTBI chronic short-term group and controls, D. Comparison of interest in new objects and old objects between the mTBI chronic short-term group and controls across genders, E. Comparison of interest in new objects and old objects between the mTBI chronic short-term and mTBI chronic long-term groups, F. Comparison of interest in new objects and old objects between the mTBI chronic short-term and mTBI chronic long-term groups and controls across genders; p<0.05*, p<0.01**, p<0.001***,p<0.0001)

When the groups were evaluated within themselves in terms of total movement in the novel object recognition test; it was determined that the mTBI chronic (CD) group moved more than the healthy control and this was statistically significant. When it was examined whether there was a significant difference between the genders, it was determined that the mTBI chronic (CD) males moved less than the females and this difference was statistically significant. At the same time, the mTBI chronic (CD) females moved statistically more on the platform than both the mTBI chronic (CD) sham control and healthy control females **(Figure 2A-B).**

When the total distance covered on the platform was evaluated in the mTBI chronic (CD), mTBI chronic (CD) sham control, and healthy control groups; no significant difference was detected between the groups. When the differences between the genders were examined, no statistically significant difference was detected **(Figure 2C-D).**

When it was examined in terms of total distance covered between the experimental groups; no statistically significant difference was detected between the mTBI chronic (CD) and mTBI chronic (CD) groups. When the differences between genders were examined, no statistically significant difference was detected between females and males **(Figure 2E-F).**

**
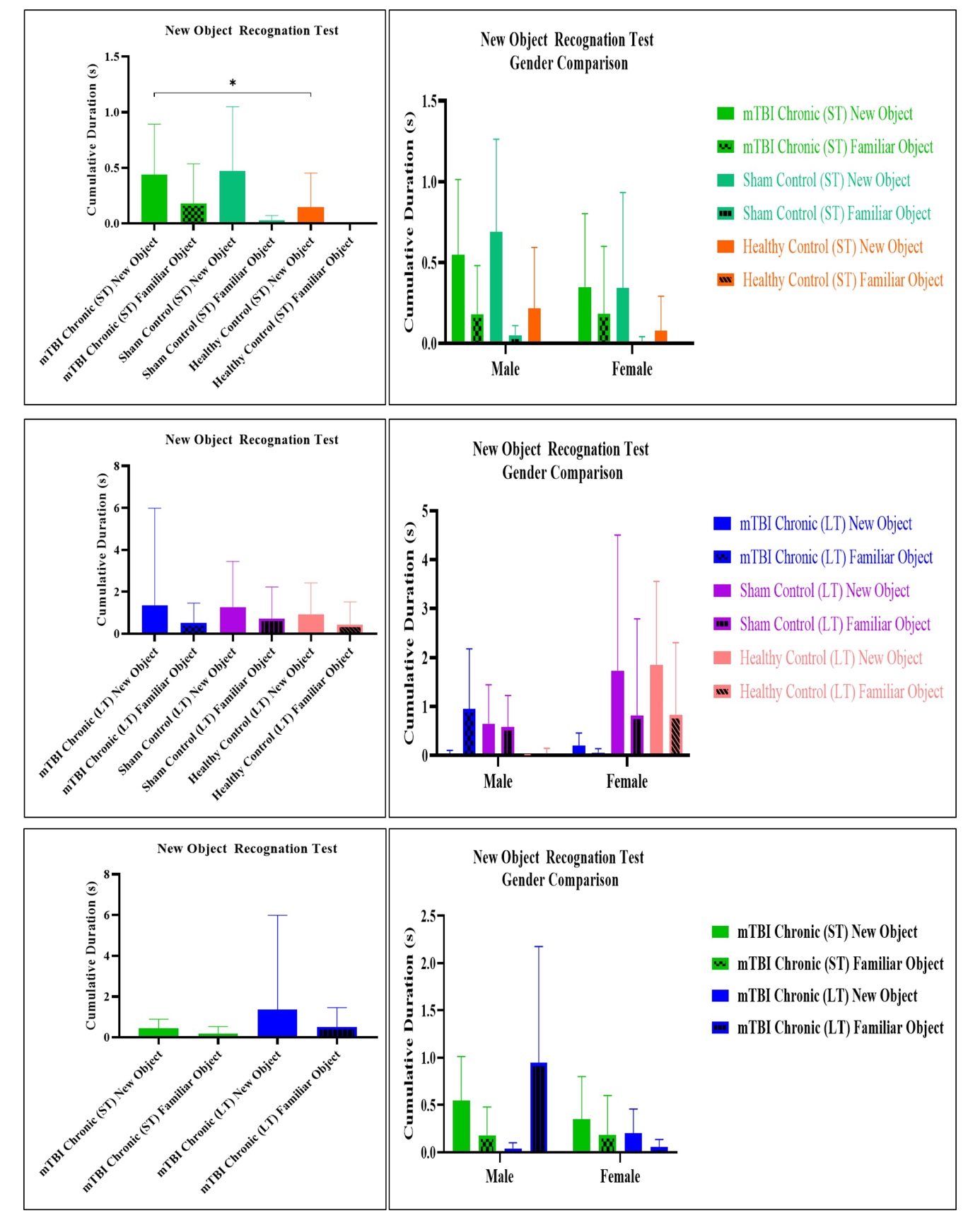
**

**Figure 2. Comparison of total distance traveled in the novel object recognition test.**

(A. Comparison of total distance traveled between mTBI chronic (ST) group and controls, B. Comparison of total distance traveled between mTBI chronic (ST) group and controls between genders, C. Comparison of total distance traveled between mTBI chronic (ST) group and controls, D. Comparison of total distance traveled between mTBI chronic (ST) group and controls between genders, E. Comparison of total distance traveled between mTBI chronic (ST) and mTBI chronic (LT) groups, F. Comparison of total distance traveled between mTBI chronic (ST) and mTBI chronic (LT) groups and controls between genders, ST: Short Term; LT: Long Term; p<0.05*, p<0.01**, p<0.001***,p<0.0001)

When the groups were evaluated within themselves in terms of the speed, they covered on the platform in the novel object recognition test; it was found that the mTBI chronic (ST) group moved more than the healthy control group and this was statistically significant. When it was examined whether there was a significant difference between the genders, it was found that the mTBI chronic (ST) males moved slower than the females and this difference was statistically significant. At the same time, it was found that the mTBI chronic (ST) females were statistically faster on the platform than both the mTBI chronic (ST) sham control and healthy control females **(Figure 3A-B).**

When the speed they covered on the platform in the mTBI chronic (UD), mTBI chronic (LT) sham control, and healthy control groups were evaluated, it was found that the mTBI chronic (LT) group moved statistically faster than the mTBI chronic (UD) sham control group. When the differences between the genders were examined, no statistically significant difference was detected **(Figure 3C-D).**

When it was examined in terms of the total speed covered between the experimental groups; No statistically significant difference was detected between the mTBI chronic (ST) and mTBI chronic (LT) groups. When the differences between the genders were examined, no statistically significant difference was detected between females and males **(Figure 3E-F).**

**
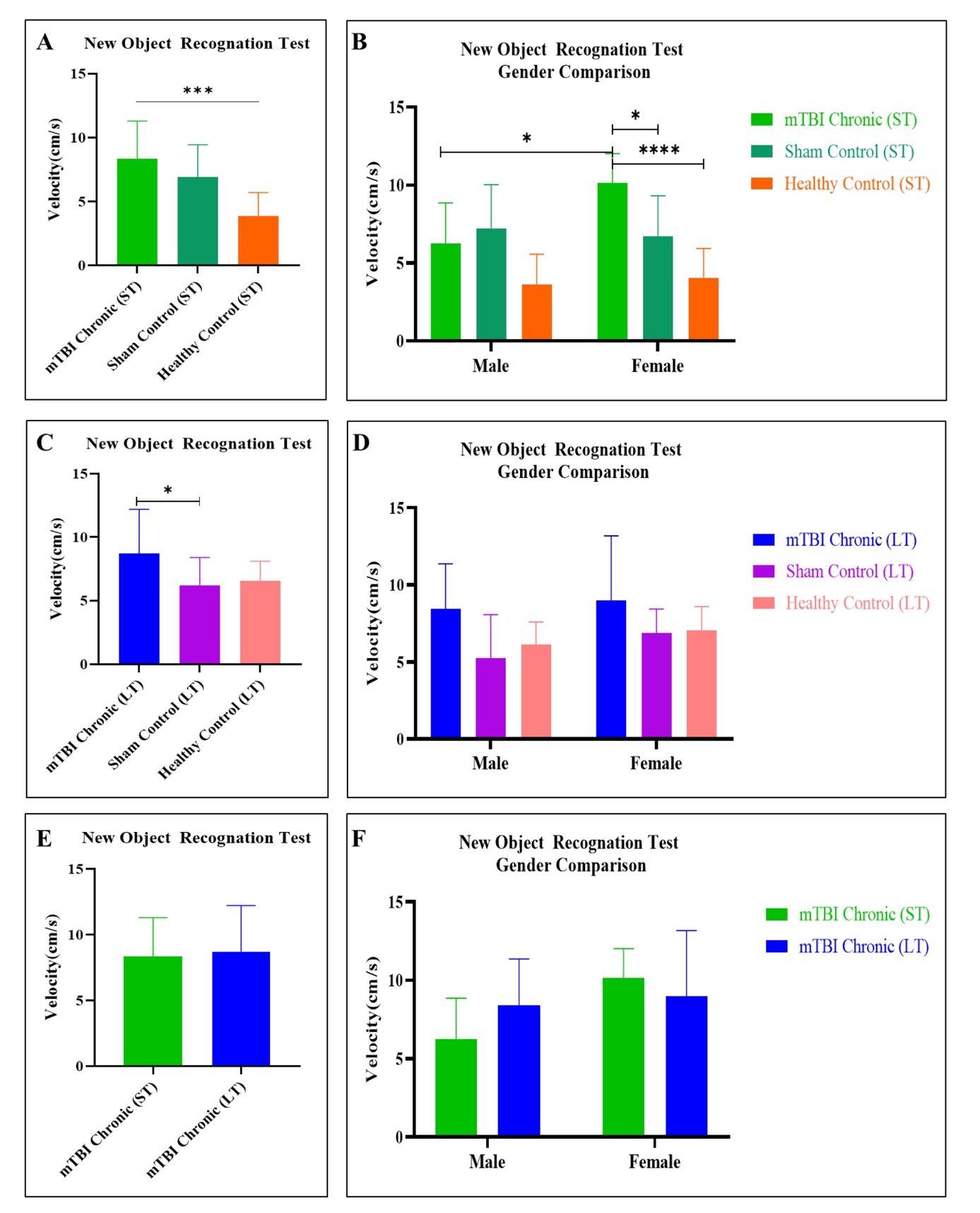
**

**Figure 3. Comparison of total speed traveled in the novel object recognition test.**

(A. Comparison of total speed traveled between mTBI chronic (ST) group and controls, B. Comparison of total speed traveled between mTBI chronic (ST) group and controls between genders, C. Comparison of total speed traveled between mTBI chronic (LT) group and controls, D. Comparison of total speed traveled between mTBI chronic (LT) group and controls between genders, E. Comparison of total speed traveled between mTBI chronic (ST) and mTBI Chronic (LT) groups, F. Comparison of total speed traveled between mTBI chronic (ST) and mTBI Chronic (LT) groups and controls between genders, ST: Short Term; LT: Long Term; p<0.05*, p<0.01**, p<0.001***,p<0.0001).

When the groups were evaluated regarding the discrimination index calculated with the data obtained in the novel object recognition test, there was no significant difference between the mTBI chronic (ST) group, the mTBI chronic (ST) sham control group, and the healthy control group. In contrast, the mTBI chronic (ST) group had the highest discrimination index. When the differences between the genders were examined, no statistically significant difference was detected. (**Figure 4A-B**). When the mTBI chronic (ST), mTBI chronic (ST) sham control, and healthy control groups were evaluated regarding the discrimination index, no statistically significant difference was detected between the groups. However, the discrimination index was the lowest in the mTBI chronic (ST) group. This finding indicates that the interest in the new object on the platform was quite low throughout the test. When the differences between the genders were examined, no statistically significant difference was detected, while the discrimination index of the mTBI chronic (ST) group was again quite low. This indicates that the low interest in the new object in the mTBI chronic (ST) group was due to men. In other words, the secondary effects of trauma are mostly seen in the mTBI chronic (LT) group of males (**Figure 4C-D**). When the experimental groups were examined in terms of discrimination index, it was not determined that the mTBI chronic (ST) group had a statistically significant discrimination index compared to the mTBI chronic (LT) group. When the differences between the genders were examined, no statistically significant difference was detected in females and males (**Figure 4E-F**).


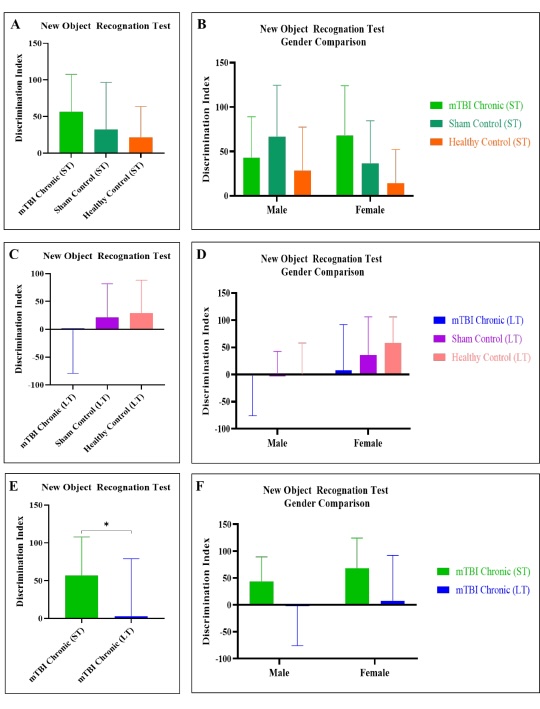
**Figure 4. Comparison of discrimination index data in a novel object recognition test.**

(A. Comparison of discrimination index data between mTBI chronic (ST) group and controls, B. Comparison of discrimination index data between mTBI chronic (ST) group and controls between genders, C. Comparison of discrimination index data between mTBI chronic (LT) group and controls, D. Comparison of discrimination index data between mTBI chronic (LT) group and controls between genders, E. Comparison of discrimination index data between mTBI chronic (ST) and mTBI chronic (LT) groups, F. Comparison of discrimination index data between mTBI chronic (ST) and mTBI chronic (LT) groups and controls between genders, ST: Short Term; LT: Long Term; p<0.05*, p<0.01**, p<0.001***,p<0.0001)

When the mTBI chronic (ST) group and mTBI chronic (ST) sham control group were evaluated in terms of the percentage spent exploring parameters with the data obtained in the novel object recognition test, the percentage spent exploring increased statistically compared to the mTBI chronic (ST) group and the healthy control group, when the differences between the genders were examined, it was determined that the females of the mTBI chronic (ST) group spent more time exploring than the healthy control females and this difference was found to be statistically significant. (**Figure 5A-B**).

When the mTBI chronic (LT), mTBI chronic (LT) sham control, and healthy control groups were evaluated regarding the time spent exploring, no statistically significant difference was found between the groups. When the differences between the genders were examined, no statistically significant difference was found (**Figure 5C-D**).

When the experimental groups were examined in terms of the time spent exploring, it was found that the mTBI chronic (ST) group spent more time exploring than the mTBI chronic (LT) group, and this difference was found to be statistically significant. When the differences between genders were examined, no statistically significant difference was detected between males and females (**Figure 5E-F**).


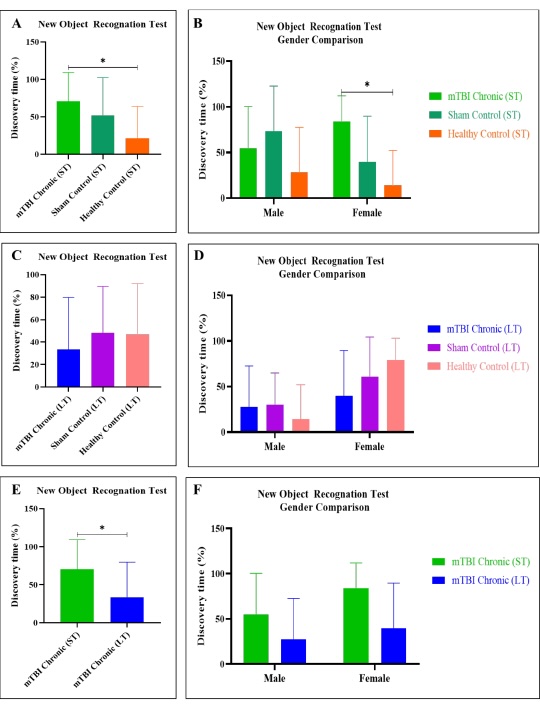
**Figure 5. Comparison of time spent exploring in a novel object recognition test.**

(A. Comparison of time spent exploring between the mTBI chronic (ST) group and controls, B. Comparison of time spent exploring between the mTBI chronic (ST) group and controls across genders, C. Comparison of time spent exploring between the mTBI chronic (LT) group and controls, D. Comparison of time spent exploring between the mTBI chronic (LT) group and controls across genders, E. Comparison of time spent exploring between the mTBI chronic (ST) and mTBI chronic (LT) groups, F. Comparison of time spent exploring between the mTBI chronic (ST) and mTBI chronic (LT) groups and controls across genders, KD: Short Term; UD: Long Term; p<0.05*, p<0.01**, p<0.001***,p<0.0001)

**1.2. Social Interaction Test Findings**

This test, which measures empathy ability related to social interaction, was subjected to two different processes to evaluate the communication of mice with each other after trauma. It was examined whether there were differences within the groups in the mice walking in the middle of cages with and without mice. Accordingly, while no significant difference was detected within the groups in the mTBI chronic (ST), mTBI chronic (ST) sham control, and healthy control groups, it was found that the interest of the mTBI chronic (ST) and mTBI chronic (ST) sham control group in the cage without mice was less than the interest of the healthy control in the cage without mice and this difference was statistically significant. As a result of the analyses made to determine the differences between the sexes, no significant difference was detected in male mice within the groups and between the groups between going to the cage with or without mice, while in females, it was found that the interest of the mTBI chronic (ST) and mTBI chronic (ST) sham control group in the cage without mice was less than the interest of the healthy control in the cage without mice and this difference was statistically significant (**Figure 6A-B**).

When it was evaluated whether there was a difference between the mTBI chronic (LT) and mTBI chronic (LT) sham control groups and healthy controls going to cages with or without mice, no significant difference was found within the groups in terms of going to cages with or without mice, and no statistically significant difference was found between the groups in terms of going to cages with or without mice. When comparisons were made between the genders, again, no statistically significant difference was found (**Figure 6C-D**). When mTBI chronic (ST) and mTBI chronic (LT) experimental groups were compared with each other, no statistically significant difference was found. Again, when it was investigated whether there were differences between the genders, no statistically significant difference was found between the genders (**Figure 6E-F**).


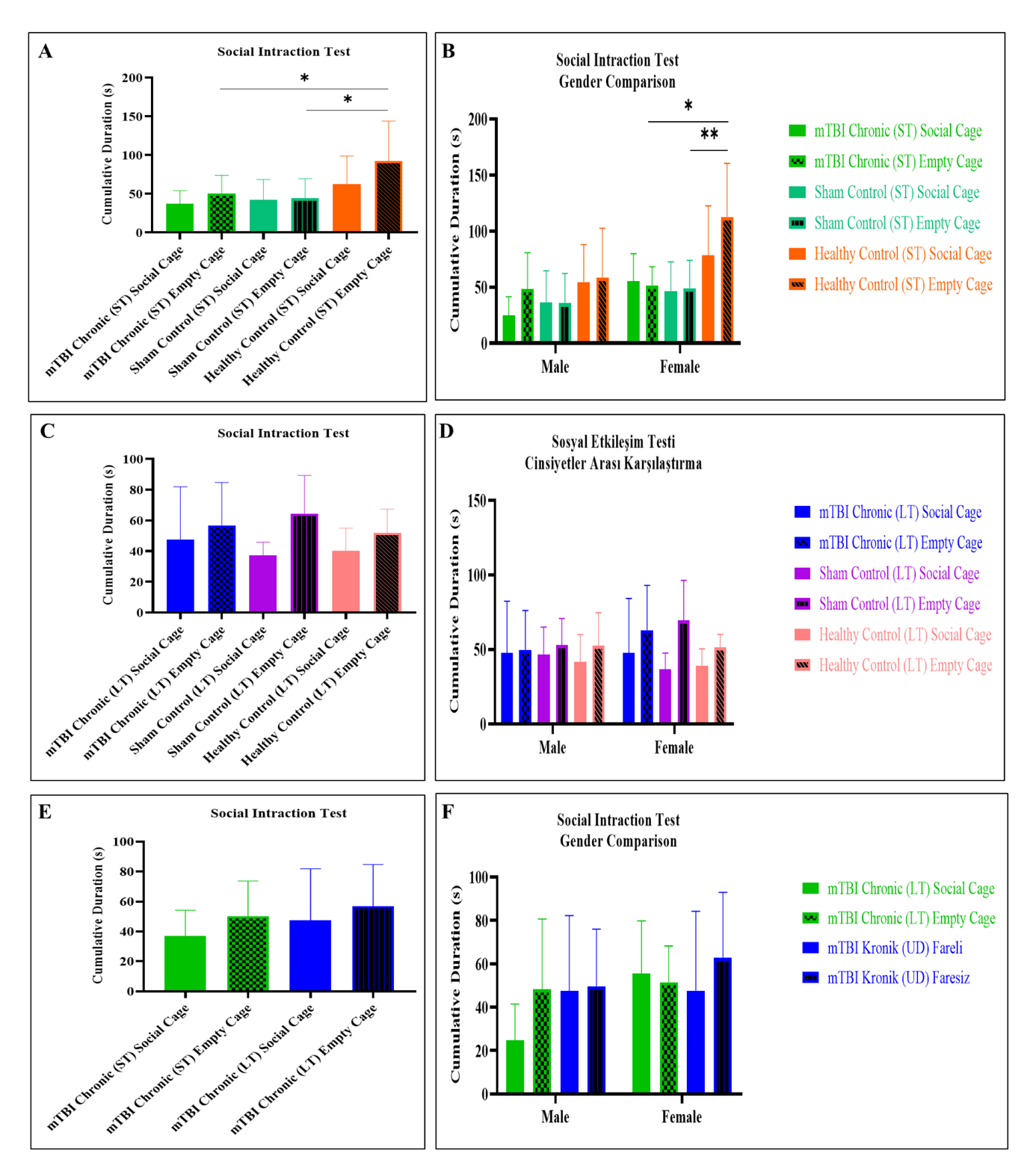
**Figure 6. Comparison of interest in cages with and without mice in the social interaction test.**

(A. Comparison of interest in the cage with mice and cage without mice between the mTBI chronic (ST) group and controls, B. Comparison of interest in the cage with mice and cage without mice between the mTBI chronic (ST) group and controls across genders, C. Comparison of interest in the cage with mice and cage without mice between the mTBI chronic (LT) group and controls, D. Comparison of interest in the cage with mice and cage without mice between the mTBI chronic (LT) group and controls across genders, E. Comparison of interest in the cage with mice and cage without mice between the mTBI chronic (ST) and mTBI Chronic (LT) groups, F. Comparison of interest in the cage with mice and cage without mice between the mTBI chronic (ST) and mTBI Chronic (LT) groups and controls across genders, CD: Short Term; UD: Long Term; p<0.05*, p<0.01**, p<0.001***,p<0.0001)

When the mTBI chronic (ST) group, mTBI chronic (ST) sham control group, and healthy control groups were compared in terms of the total distance covered by the mice during the social interaction test, it was determined that the mTBI chronic (ST) group and mTBI chronic (ST) sham control group covered statistically significant and more distance than the healthy control. When the differences between the genders were evaluated, it was seen that in male mice, the mTBI chronic (ST) sham control group covered statistically significant and more distance than the healthy control; in females, the mTBI chronic (ST) group and mTBI chronic (KD) sham group covered statistically significant and more distance than the healthy control (**Figure 7A-B**).

When the mTBI chronic (LT), mTBI chronic (LT) sham control and healthy control groups were evaluated regarding the total distance covered, no significant difference was detected between the groups. When the differences between the genders were evaluated, no statistically significant difference was detected between the male and female mice (**Figure 7C-D**).

When the experimental groups were evaluated among themselves, no significant difference was detected in the mTBI chronic (ST) and mTBI chronic (LT) groups in terms of total distance traveled. When the differences between the sexes were evaluated, it was found that male mice traveled more distance than the mTBI chronic (ST) group and mTBI chronic (LT) group, and the females of the mTBI chronic (ST) group traveled more distance than the males. It was determined that these differences were statistically significant (**Figure 7E-F**).


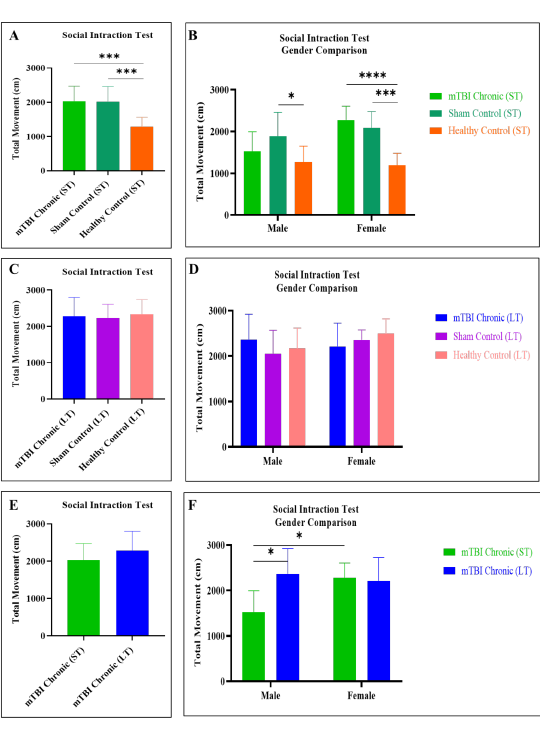
**Figure 7. Comparison of total distance traveled in the social interaction test.**

(A. Comparison of total distance traveled between mTBI chronic (ST) group and controls, B. Comparison of total distance traveled between mTBI chronic (ST) group and controls between genders, C. Comparison of total distance traveled between mTBI chronic (LT) group and controls, D. Comparison of total distance traveled between mTBI chronic (LT) group and controls between genders, E. Comparison of total distance traveled between mTBI chronic (ST) and mTBI chronic (LT) groups, F. Comparison of total distance traveled between mTBI chronic (ST) and mTBI chronic (LT) groups and controls between genders, KD: Short Term; UD: Long Term; p<0.05*, p<0.01**, p<0.001***,p<0.0001)

During the social interaction test, when the mTBI chronic (ST) group, mTBI chronic (ST) sham control group, and healthy control groups were compared in terms of the speed of the mice, it was found that the mTBI chronic (ST) group and mTBI chronic (ST) sham control group were statistically significantly faster than the healthy control. When the differences between the genders were evaluated, it was found that the mTBI chronic (ST) sham control group was statistically significantly faster than the healthy control in male mice; and in female mice, the mTBI chronic (ST) group and mTBI chronic (ST) sham group were statistically significantly faster than the healthy control and behaved faster on the platform (**Figure 8A-B**).

When the speed of the mTBI chronic (LT), mTBI chronic (LT) sham control, and healthy control groups on the platform was evaluated, no significant difference was detected between the groups. When the differences between the genders were evaluated, no statistically significant difference was detected between male and female mice (**Figure 8C-D**).

When the experimental groups were evaluated in terms of the speed they traveled on the platform, no significant difference was detected in the mTBI chronic (ST) and mTBI chronic (LT) groups. When the differences between the sexes were evaluated, it was found that male mice moved faster than the mTBI chronic (ST) group and mTBI chronic (LT) group, and the females of the mTBI chronic (ST) group were faster on the platform than the males. It was determined that these differences were statistically significant (**Figure 8E-F**).

**
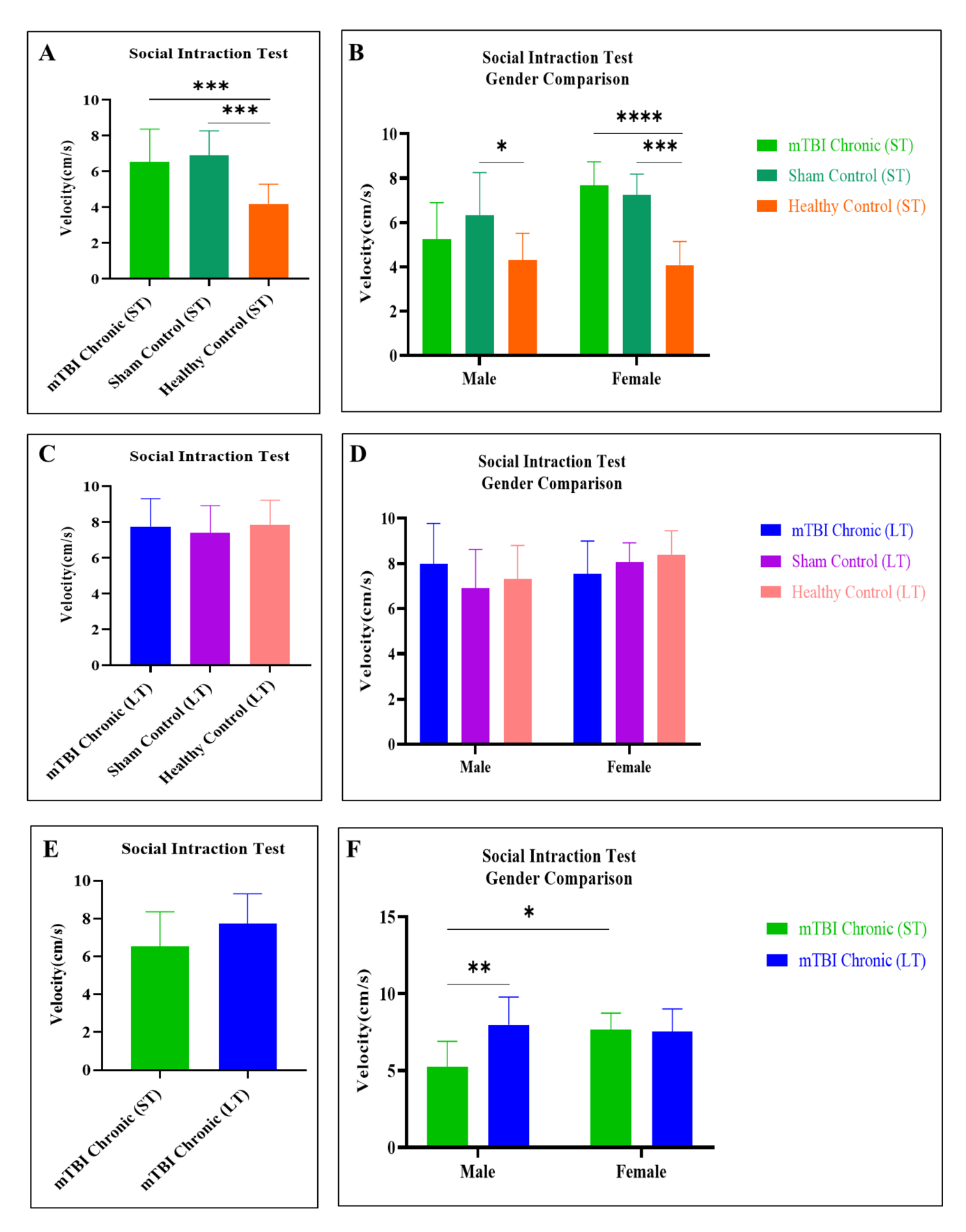
Figure 8. Comparison of total speed traveled in the social interaction test.**

(A. Comparison of total speed traveled between mTBI chronic (ST) group and controls, B. Comparison of total speed traveled between mTBI chronic (ST) group and controls between genders, C. Comparison of total speed traveled between mTBI chronic (LT) group and controls, D. Comparison of total speed traveled between mTBI chronic (LT) group and controls between genders, E. Comparison of total speed traveled between mTBI chronic (ST) and mTBI Chronic (LT) groups, F. Comparison of total speed traveled between mTBI chronic (ST) and mTBI Chronic (LT) groups and controls between genders, ST: Short Term; LT: Long Term; p<0.05*, p<0.01**, p<0.001***,p<0.0001).

**1.3 Tail Suspension Test Findings**

In the tail suspension test, where the post-traumatic anxiety and the time spent sedentary were measured, when the time spent sedentary was evaluated among the mTBI Chronic (ST) group, the mTBI Chronic (ST) sham control group, and the healthy control groups, it was found that the mTBI chronic (ST) group moved less than the healthy control. No significant difference was found between the genders (**Figure 9A-B**).

When the time spent sedentary was evaluated among the mTBI Chronic (LT) group, the mTBI Chronic (LT) sham control group, and the healthy control groups, no significant difference was found between the groups. When the differences were evaluated between the genders, no statistically significant difference was found (**Figure 9C-D**).

When the experimental groups were compared among themselves, no statistically significant difference was found between the mTBI chronic (ST) and mTBI chronic (LT) groups. When the changes between the genders were evaluated, no statistically significant difference was found (**Figure 9E-F**).


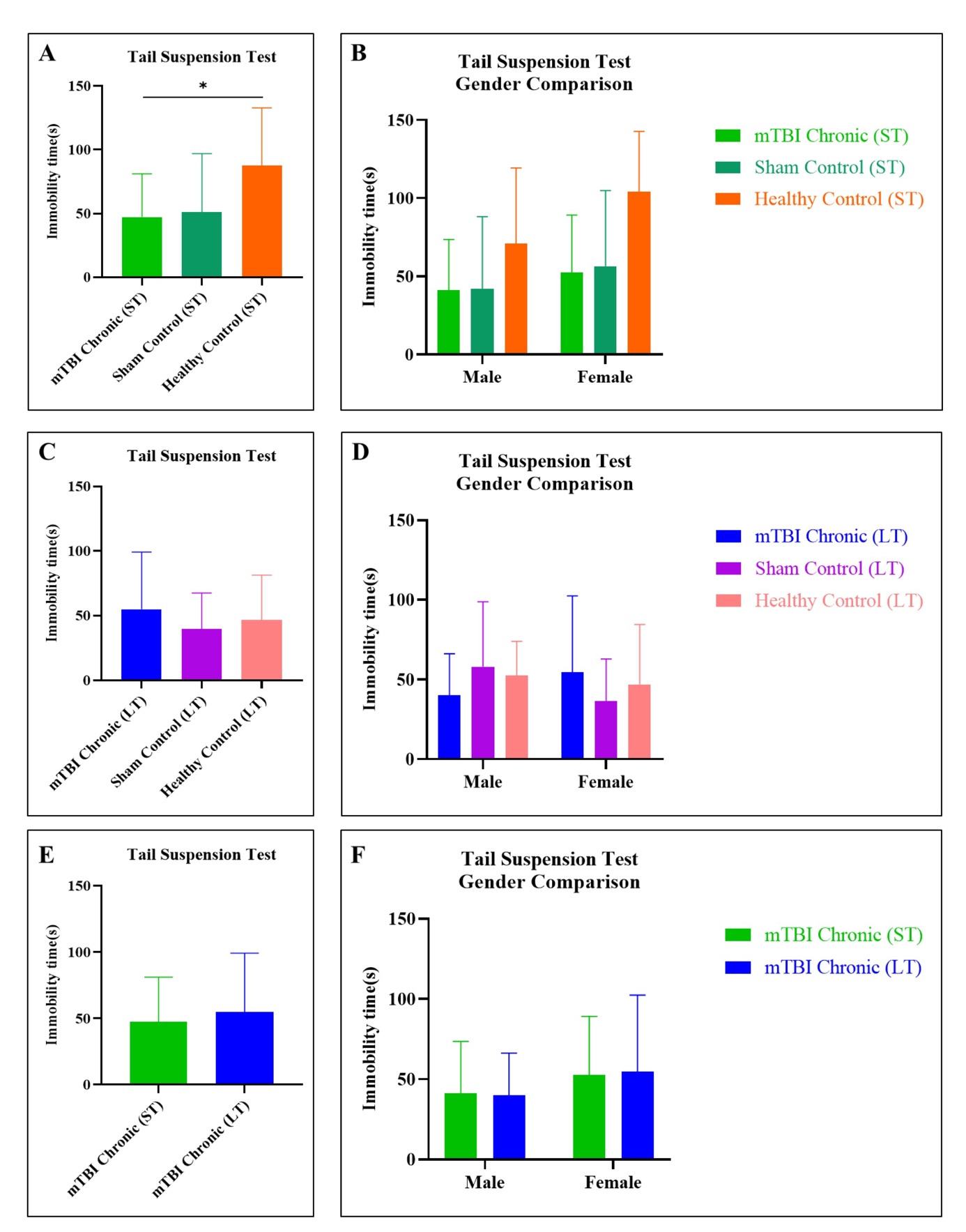
**Figure 9. Comparison of total immobile time in tail suspension test.**

(A. Comparison of total sedentary time between the mTBI chronic (ST) group and controls, B. Comparison of total sedentary time between the mTBI chronic (ST) group and controls between genders, C. Comparison of total sedentary time between the mTBI chronic (LT) group and controls, D. Comparison of total sedentary time between the mTBI chronic (LT) group and controls between genders, E. Comparison of total sedentary time between the mTBI chronic (CD) and mTBI Chronic (ST) groups, F. Comparison of total sedentary time between the mTBI chronic (ST) and mTBI Chronic (LT) groups and controls between genders, KD: Short Term; UD: Long Term; p<0.05*, p<0.01**, p<0.001***,p<0.0001).

**1.4 Marble Test Findings**

In the marble test, also known as the marble burying test, when the number of marbles left by the mice buried in the cage was calculated, no statistically significant difference was found between the mTBI Chronic (ST) group, the mTBI Chronic (ST) sham control group, and the healthy control groups. When the changes between the genders were examined, no significant difference was found between the groups (**Figure 10A-B**).

No significant difference was found between the mTBI Chronic (LT) group, the mTBI Chronic (LT) sham control group, and the healthy control group. When the changes between the genders were examined, no significant difference was found again (**Figure 10 C-D**).

When the experimental groups were evaluated within themselves, no significant difference was found between the mTBI Chronic (ST) and mTBI Chronic (LT) groups. No statistically significant difference was found between the genders (**Figure 10E-F**).


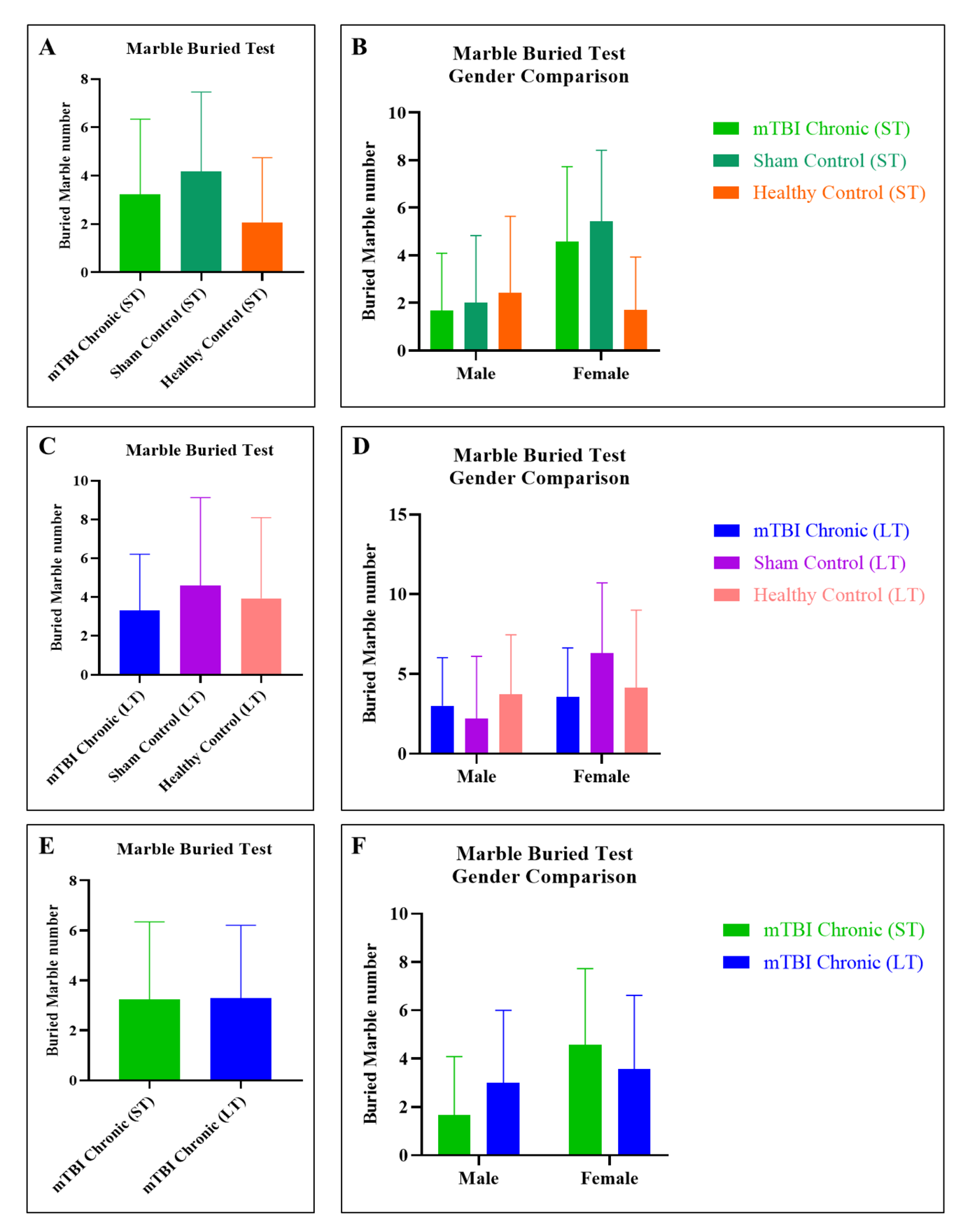
**Figure 10. Comparison of total number of embedded marbles in marble test.**

(A. Comparison of total buried marble numbers between mTBI chronic (ST) group and controls, B. Comparison of total buried marble numbers between mTBI chronic (ST) group and controls between genders, C. Comparison of total buried marble numbers between mTBI chronic (LT) group and controls, D. Comparison of total buried marble numbers between mTBI chronic (LT) group and controls between genders, E. Comparison of total buried marble numbers between mTBI chronic (ST) and mTBI Chronic (LT) groups, F. Comparison of total buried marble numbers between mTBI chronic (ST) and mTBI Chronic (LT) groups and controls between genders, KD: Short Term; UD: Long Term; p<0.05*, p<0.01**, p<0.001***,p<0.0001).

**1.5 Open Field Test Findings**

In the open field test where anxiety and claustrophobia were evaluated, the post-traumatic experimental groups and controls were examined in terms of the time spent in the central and peripheral areas. In this test, where healthy mice were expected to wander frequently in the central area and traumatic mice in the peripheral area, it was determined that the time spent in the peripheral area in the mTBI chronic (ST) group was less than the time spent in the central area and this finding was statistically significant. Although the same finding was found in the mTBI chronic (ST) sham control and healthy control groups, these findings were not statistically significant. When the differences between the genders were evaluated, it was seen that both males and females of the mTBI chronic (ST), mTBI chronic (ST) sham control, and healthy control groups spent more time in the central area (**Figure 11A-B**).

When the times spent in the peripheral area and the central area of ​​the mTBI chronic (LT), mTBI chronic (LT) sham control, and healthy control groups were compared, It was determined that the mTBI chronic (LT) group spent more time in the central area than the peripheral area and this finding was statistically significant. Although the same findings were obtained in the mTBI chronic (LT) sham control group and the healthy control group, they were not statistically significant. When the differences between the genders were evaluated, it was seen that both males and females in the mTBI chronic (LT), mTBI chronic (LT) sham control and healthy control groups spent more time in the central area (**Figure 11 C-D**).

When the mTBI chronic (ST) and mTBI chronic (LT) experimental groups were compared among themselves; it was observed that the time spent in the peripheral area was less than the time spent in the central area in both the mTBI chronic (ST) and mTBI chronic (LT) groups; the time spent in the peripheral area by the mTBI chronic (ST) group was less than the time spent in the central area by the mTBI chronic (LT) group. It was also found that the time spent in the central area by the mTBI chronic (ST) group was more than the time spent in the peripheral area by the mTBI chronic (LT) group and the findings were statistically significant. When the differences between the genders were evaluated, it was seen that both males and females of the mTBI chronic (ST), mTBI chronic (ST) sham control, and healthy control groups spent more time in the central area (**Figure 11 E-F**).


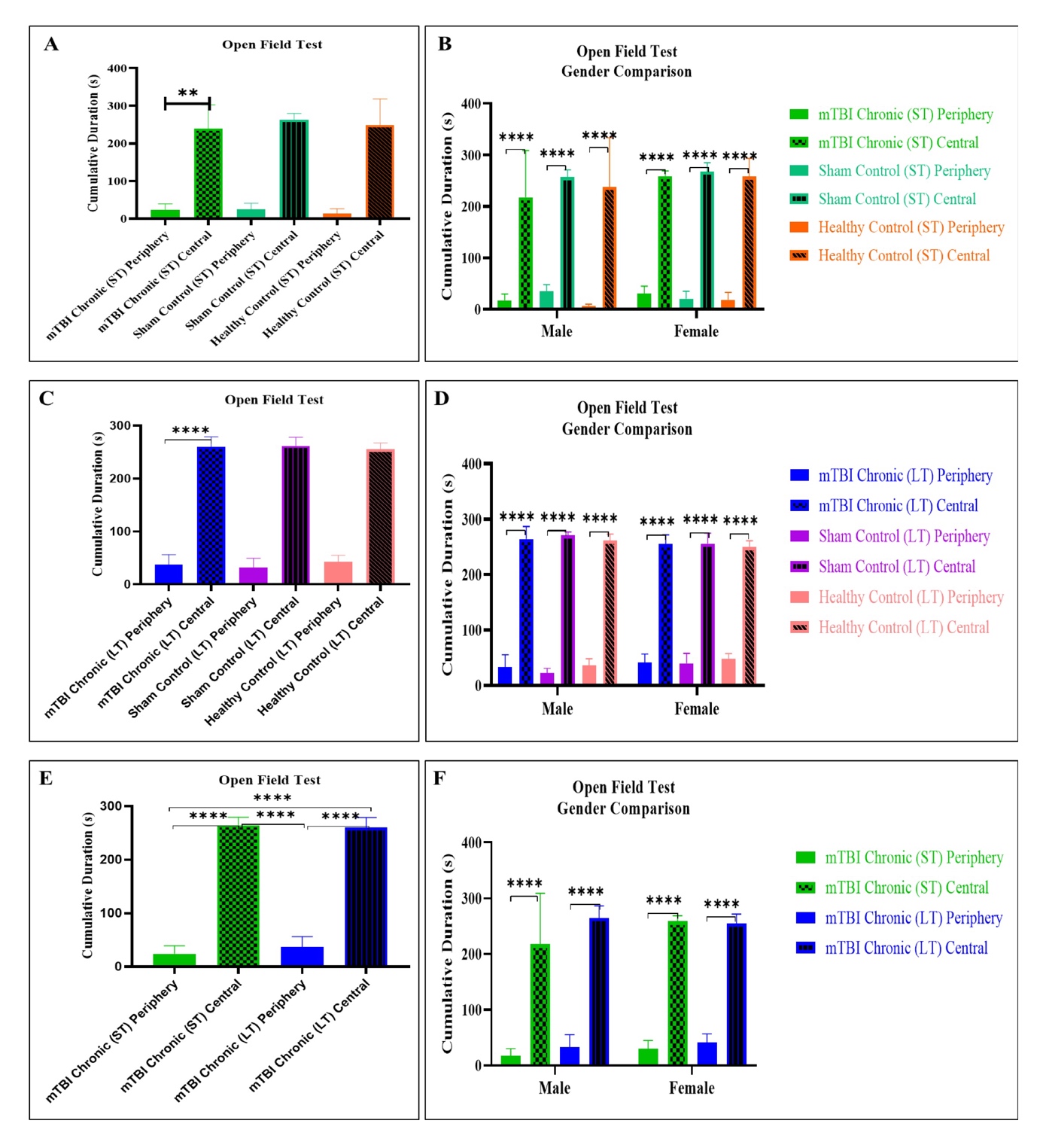
**Figure 11. Comparison of time spent in the peripheral and central areas in the open field test.**

(A. Comparison of the time spent in the peripheral field and central field between the mTBI chronic (ST) group and controls, B. Comparison of the time spent in the peripheral field and central field between the mTBI chronic (ST) group and controls between genders, C. Comparison of the time spent in the peripheral field and central field between the mTBI chronic (LT) group and controls, D. Comparison of the time spent in the peripheral field and central field between the mTBI chronic (LT) group and controls between genders, E. Comparison of the time spent in the peripheral field and central field between the mTBI chronic (ST) and mTBI chronic (LT) groups, F. Comparison of the time spent in the peripheral field and central field between the mTBI chronic (ST) and mTBI chronic (LT) groups and controls between genders, KD: Short Term; UD: Long Term; p<0.05*, p<0.01**, p<0.001***,p<0.0001)

When the distance covered on the platform during the open field test was evaluated between the experimental groups; it was determined that the mTBI chronic (ST) and mTBI chronic (ST) sham control groups covered more distance than the healthy control group and these findings were statistically significant. When the differences between the genders were evaluated; it was determined that the mTBI chronic (ST) males covered statistically significant and less distance than the mTBI chronic (ST) females. At the same time, it was observed that the mTBI chronic (KD) females covered more distance than the healthy control group females and the mTBI chronic (ST) sham control females covered more distance than the healthy control (**Figure 12A-B**).

When the mTBI chronic (LT), mTBI chronic (LT) sham control and healthy control groups were evaluated in terms of the distance covered during the open field test, no statistically significant difference was detected. When the differences between the genders were evaluated, no significant difference was detected between the groups and in terms of females and males (**Figure 12C-D**).

When mTBI chronic (ST) and mTBI chronic (LT) experimental groups were compared with each other, no statistically significant difference was found between the groups. When the differences between the genders were examined, it was seen that only mTBI chronic (ST) men covered less distance than mTBI chronic (LT) men (**Figure 12E-F**).


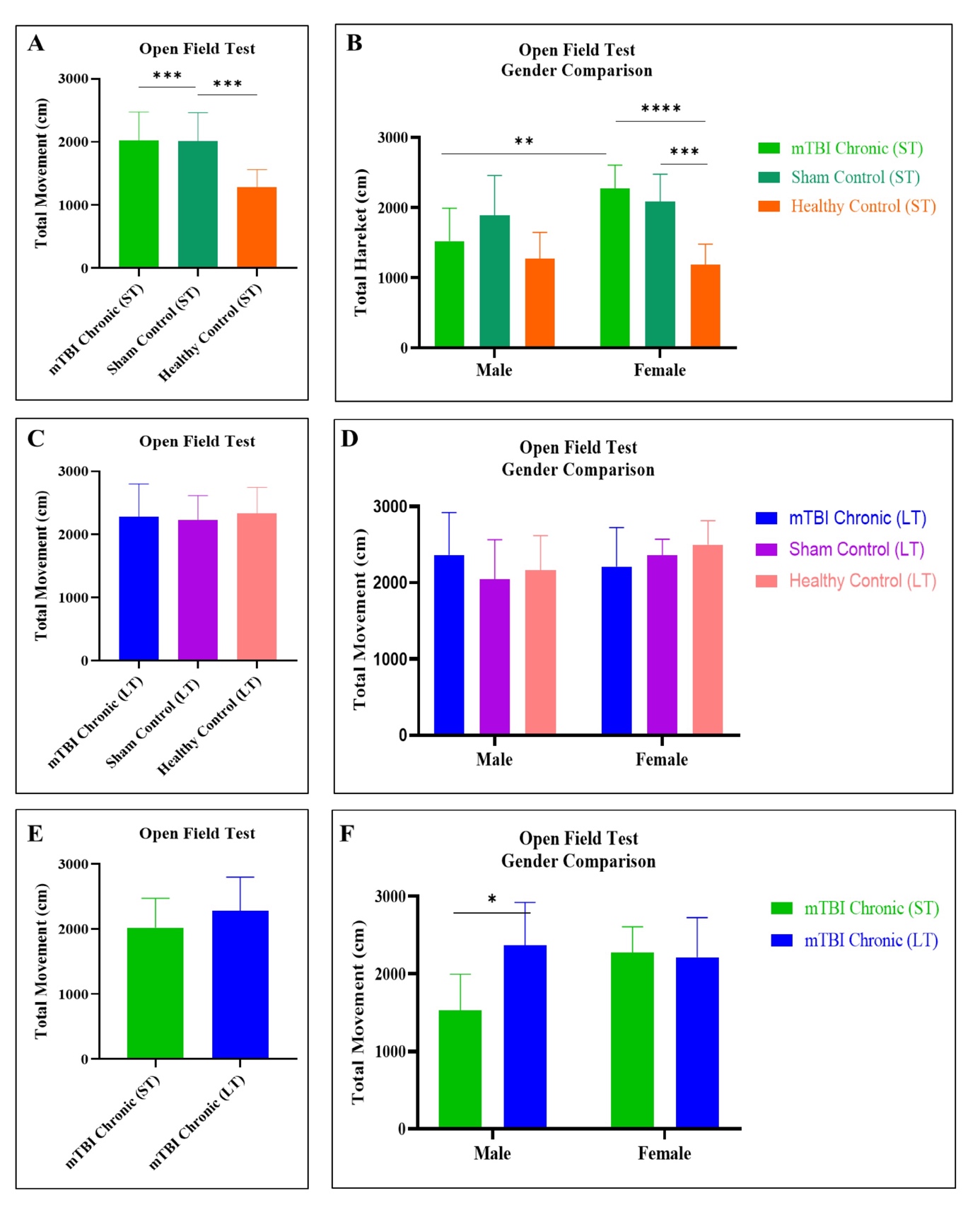
**Figure 12. Comparison of total distance covered in open field test.**

(A. Comparison of total distance traveled between mTBI chronic (ST) group and controls, B. Comparison of total distance traveled between mTBI chronic (ST) group and controls between genders, C. Comparison of total distance traveled between mTBI chronic (LT) group and controls, D. Comparison of total distance traveled between mTBI chronic (LT) group and controls between genders, E. Comparison of total distance traveled between mTBI chronic (ST) and mTBI chronic (LT) groups, F. Comparison of total distance traveled between mTBI chronic (ST) and mTBI chronic (LT) groups and controls between genders, KD: Short Term; UD: Long Term; p<0.05*, p<0.01**, p<0.001***,p<0.0001)

When the speeds covered on the platform during the open field test were evaluated between the experimental groups; mTBI chronic (ST) and mTBI chronic (ST) sham control groups were found to be faster than the healthy control group and these findings were statistically significant. When the differences between the sexes were evaluated; mTBI chronic (ST) males were found to be statistically significant and covered less distance than mTBI chronic (ST) females. In addition, mTBI chronic (ST) sham control males were found to be faster than healthy controls. At the same time, mTBI chronic (ST) females were found to be faster than healthy control females and mTBI chronic (ST) sham control females were found to be faster than healthy controls (**Figure 13A-B**).

When mTBI chronic (LT), mTBI chronic (LT) sham control and healthy control groups were evaluated in terms of speed covered during the open field test, no statistically significant difference was found. When the differences between genders were evaluated, no significant difference was found between the groups and in terms of females and males (**Figure 13C-D**). When the mTBI chronic (ST) and mTBI chronic (LT) experimental groups were compared with each other, no statistically significant difference was found between the groups. When the differences between genders were examined, it was seen that only mTBI chronic (ST) males were slower than mTBI chronic (LT) males (**Figure 13 E-F**).


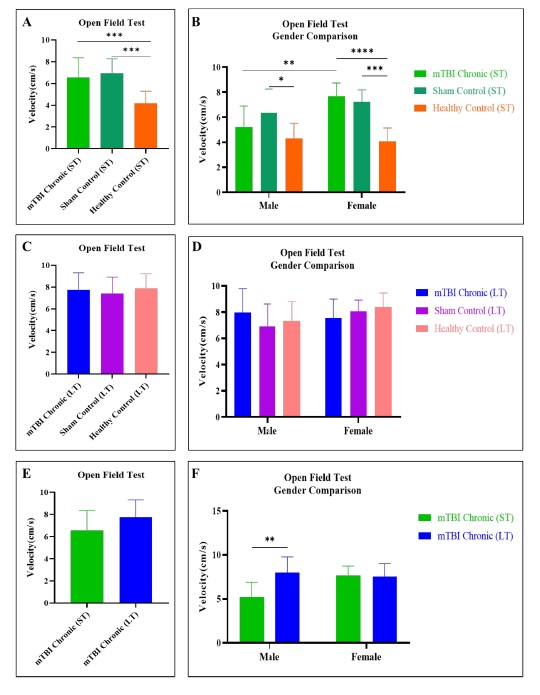
**Figure 13. Comparison of total speed covered in open field test.**

(A. Comparison of total speed traveled between mTBI chronic (ST) group and controls, B. Comparison of total speed traveled between mTBI chronic (ST) group and controls between genders, C. Comparison of total speed traveled between mTBI chronic (LT) group and controls, D. Comparison of total speed traveled between mTBI chronic (LT) group and controls between genders, E. Comparison of total speed traveled between mTBI chronic (ST) and mTBI Chronic (LT) groups, F. Comparison of total speed traveled between mTBI chronic (ST) and mTBI Chronic (LT) groups and controls between genders, KD: Short Term; UD: Long Term; p<0.05*, p<0.01**, p<0.001***,p<0.0001).

In general, behavioral experiments were evaluated in their entirety in traumatized mTBI chronic (ST) and mTBI chronic (LT) groups, and phenotypic findings consistent with traumatized individuals were not obtained through behavioral experiments. The reason for this may be that secondary neurodegenerative findings after trauma manifest themselves at later ages, and these findings did not occur during the periods when behavioral experiments were conducted (for mTBI chronic (ST): 3 months; for mTBI chronic (LT): 5 months).
